# Exploration of Regulatory Elements, MicroRNAs, and Copy Number Variation in Urogenital Chlamydia Reinfection in African American Women

**DOI:** 10.3390/ijms27125410

**Published:** 2026-06-16

**Authors:** Hemant K. Tiwari, Sandeep Chowdary Vejandla, Ihsan Buker, Mengchen Ding, Vinodh Srinivasasainagendra, Amit Patki, Kanupriya Gupta, Caren Weinhouse, William M. Geisler

**Affiliations:** 1 Department of Biostatistics, School of Public Health, University of Alabama at Birmingham, Birmingham, AL 35294, USA; vsc4u@uab.edu (S.C.V.); iebuker@uab.edu (I.B.); mding@uab.edu (M.D.); vinodh@uab.edu (V.S.); apatki@uab.edu (A.P.); 2 Department of Medicine, Division of Infectious Diseases, University of Alabama at Birmingham, Birmingham, AL 35294, USA; kanupriyagupta@uabmc.edu (K.G.); wgeisler@uabmc.edu (W.M.G.); 3 Oregon Institute of Occupational Health Sciences, Oregon Health & Science University, Portland, OR 97239, USA; weinhous@ohsu.edu

**Keywords:** *Chlamydia trachomatis*, reinfection, regulatory elements, microRNAs, copy number variation

## Abstract

Host genetic susceptibility to urogenital *Chlamydia trachomatis* (*Ct*) reinfection remains poorly understood. Coding variants identified in prior genome-wide association studies (GWAS) explained only a small fraction of the risk of reinfection. Our goal in this study was to characterize whether more risk would be captured by sequence variation that traditional GWAS insufficiently captures. Specifically, we evaluated the risk attributable to SNPs present in regulatory, non-coding regions; post-transcriptional regulation by microRNAs (miRNAs) that may depend on sequence variation in either the miRNA or the target mRNA; and copy number variants (CNVs). We analyzed GWAS data from African American women with or without documented urogenital *Ct* reinfection. Fine mapping and independent association analyses identified 30 unique index single-nucleotide polymorphisms (iSNPs), which were expanded to variants in linkage disequilibrium. Regulatory annotation was performed using HaploReg, RegulomeDB, FORGEdb, rSNPBase, and GTEx. We examined whether genes identified in the *Ct* reinfection GWAS are targeted by known *Ct* infection–associated microRNAs using curated databases. Genome-wide CNV calling was conducted using SNP intensity data, followed by stringent quality control and gene-level association testing. Functional annotation prioritized 7 SNPs with strong regulatory evidence, with stringent criteria for regulatory relevance, using HaploReg, RegulomeDB, FORGEdb, and rSNPBase. The strongest signals were observed at the *CHIT1* locus, where multiple intronic variants (including rs2486963 and rs2244385) overlapped regulatory chromatin, altered transcription factor binding motifs, and acted as *cis*-expression quantitative trait loci for *CHIT1* in whole blood. Additional regulatory variants were identified near *TDRP*, *ERICH1*, and *DLGAP1*, showing tissue-specific regulatory effects. MicroRNA analysis revealed extensive post-transcriptional targeting of *SOCS6* and *SULF1*, while *CHIT1* showed no curated *Ct*-associated miRNA interactions. CNV analysis identified 5775 high-confidence events, with nominal gene-level associations observed for *ATAD3A*, *CARD14*, *TMEM240*, and *ZNF140*. These results indicate that a greater fraction of the susceptibility to urogenital *Ct* reinfection may be driven by genetic variation affecting immune and epithelial pathways rather than protein-coding changes.

## 1. Introduction

To date, more than 45,000 genome-wide association studies (GWAS) have been published [1]. Approximately 43% of trait-associated single-nucleotide polymorphisms (SNPs) map to intergenic regions, while about 45% are located within introns [2]. When additional non-coding elements, including promoters, untranslated regions (UTRs), and non-coding RNAs, are considered, over 90% of trait-associated SNPs reside outside protein-coding sequences, leaving only a small fraction (~7%) within coding regions [2]. Collectively, these findings indicate that most GWAS-identified variants lie outside protein-coding regions and, although their functions are often not well characterized, they are likely to exert regulatory effects.

Recently, we reported the first genome-wide association study (GWAS) of urogenital *Chlamydia trachomatis* (*Ct*) reinfection in African American women [3]. Our study identified 19 independent intronic or intergenic SNPs associated with *Ct* reinfection [3]. Additionally, fine-mapping analysis revealed 13 SNPs linked to susceptibility, each with a posterior probability ≥0.2 [3]. In silico chromatin interaction and eQTL analyses further implicated several genes, including *CHIT1*, *CHI3L1*, and *ADORA1* on chromosome 1; *TDRP*, *FBXO25*, and *SULF1* on chromosome 8; and *SOCS6* on chromosome 18. In contrast, *ALK* on chromosome 2 was the only locus without support from chromatin interaction or eQTL evidence [3].

Notably, the majority of SNPs with *p*-values < 10^−2^ were located in non-coding regions, with 54.27% classified as intergenic, 37.21% intronic, and 5.93% intronic within non-coding RNAs; only a small proportion mapped to exonic (0.51%) or ncRNA exonic (0.33%) regions [3]. This pattern became more pronounced among the most significant associations (*p* < 10^−5^), for which no exonic SNPs were observed; instead, variants were almost exclusively intergenic (22.58%), intronic (51.61%), or ncRNA intronic (22.58%) [3]. Collectively, these findings suggest that non-coding regulatory variation may contribute substantially to genetic susceptibility to *Ct* reinfection, underscoring the need for systematic functional characterization of these loci.

In addition to single-nucleotide variants, copy number variations (CNVs) represent a distinct form of genomic variation that can substantially influence host susceptibility to infectious diseases [4,5]. Characterizing CNVs is therefore essential in infectious disease research, as alterations in gene dosage and genomic structure can directly affect immune function and host–pathogen interactions [6,7], revealing genetic effects that may not be captured by SNP-only association analyses [4]. CNVs refer to duplications or deletions of large DNA segments in the genome, affecting around 10% of the total genetic material. These variations, which may involve different numbers of repeats, can range from 1 kilobase (kb) to 3 megabases (Mb) [6,8,9]. CNVs that overlap in multiple individuals form regions known as copy number variable regions (CNVRs) [8].

Variations in CNVs can also affect the function of immune response genes, potentially making individuals more susceptible to infections, depending on whether the CNV occurs in innate/adaptive immune-related genomic regions. For example, variable copy number of the chemokine gene *CCL3L1* has been associated with altered susceptibility to and progression of HIV infection, showing how dosage changes can modulate host defense [10,11]. In response to pathogen infections, CNVs can be either advantageous or disadvantageous, depending on the number of gene copies a person carries [12]. These CNVs can affect the expression of immune-related genes, particularly those involved in antigen recognition [9]. Specifically, regions of the genome that encode immune receptors, such as the immunoglobulin and T cell receptor genes, which are crucial for recognizing a wide range of antigens, are particularly prone to CNVs [9,12,13].

Although copy number variation (CNV) affecting NOD-like receptor (NLR) genes has not yet been directly investigated in relation to *Ct* infection, substantial evidence indicates that *NOD1* and *NOD2* are critical for intracellular recognition of chlamydial peptidoglycan and for regulating downstream inflammatory responses during infection [14,15]. Moreover, functional polymorphisms in *NOD1* have been shown to influence susceptibility to *Ct* infection and disease severity, including the risk of tubal factor infertility [16]. As a family, NLRs function as cytosolic pattern-recognition receptors (PRRs) that detect pathogen-associated molecular patterns (PAMPs) and initiate innate immune signaling pathways essential for host defense [17,18,19,20,21,22,23,24,25,26].

Similarly, copy-number variation in antimicrobial-peptide (β-defensin) genes and in Fragment crystallizable receptor (Fc-receptor) loci (e.g., *DEFB* and *FCGR3B*) has been linked to differences in pathogen control, immune activation, and clinical outcome in infectious and immune-mediated diseases [27,28]. Because CNVs often differ in frequency between populations and can underlie substantial phenotypic variance, mapping their contributions can clarify mechanisms of susceptibility, point to molecular targets (or biomarkers) for prevention and therapy, and improve risk prediction beyond SNP-based approaches [29]. Collectively, these observations identify NOD-like receptors or β-defensin genes as biologically compelling yet largely unexplored candidates for CNV-based studies of *Ct* reinfection susceptibility.

Increasing evidence implicates host microRNAs (miRNAs) in regulating immune responses and disease outcomes during *Ct* reinfection. Experimental studies show that reinfection induces distinct miRNA profiles linked to inflammation and tissue remodeling, with miR-378b and miR-135a influencing bacterial clearance, immunopathology, and T cell–mediated responses [30,31,32]. Human studies further support altered circulating miRNA signatures in reinfection [33]. Accordingly, we examined whether genes identified in our *Ct* reinfection GWAS [3] are targeted by infection-associated miRNAs. We speculate that sequence variation in phenotypically relevant miRNA or their mRNA targets (including in non-coding, intronic sequence) may impact these regulatory circuits, altering reinfection risk.

To date, no studies have systematically evaluated the regulatory consequences of SNPs, miRNA-targeted genes, and CNVs in the context of *Ct* reinfection. In this study, we addressed this gap by investigating the regulatory potential of SNPs identified from our prior *Ct* reinfection GWAS [3], analyzing known *Ct* reinfection-associated genes targeted by microRNAs, and characterizing genome-wide CNVs to assess their role in *Ct* reinfection. Together, these analyses provide an exploratory framework for identifying regulatory and structural genetic mechanisms that may contribute to *Ct* reinfection susceptibility.

## 2. Results

**Figure 1:**
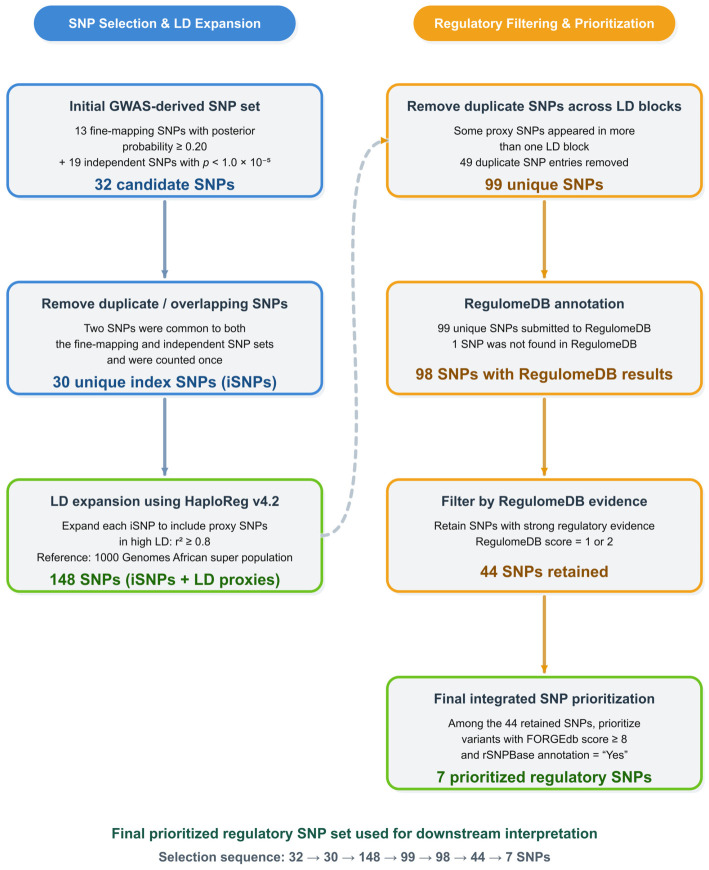
Single Nucleotide Polymorphisms (SNPs) selection and prioritization pipeline. Stepwise filtering of *Chlamydia trachomatis* reinfection GWAS-derived SNPs and regulatory SNP prioritization.

A schematic of the stepwise framework used to prioritize regulatory SNPs identified in the *Ct* reinfection GWAS, is depicted in Figure 1 [3]. This pipeline includes systematic SNP selection, followed by functional annotation and multi-layered prioritization to identify variants with potential regulatory relevance.

*Selection of SNPs to investigate regulatory consequences.* Supplementary Table S1 provides 13 significant fine-mapping SNPs with Posterior Probability (PP) greater than or equal to 0.20, and Supplementary Table S2 contains 19 significant independent SNPs from *Ct* reinfection GWAS with *p*-value < 1.0 × 10^−5^ identified in Tiwari et al. [3]. There were two SNPs common to both fine-mapping and independent SNP sets, namely, rs2486961 (*CHIT1*) and rs111891071 (*ALK*). We used 30 unique SNPs for further analysis and called them index SNPs (iSNPs).

*Investigation of the regulatory potential of significant GWAS SNPs*. We used HaploReg v4.2 [34], to annotate the regulatory potential of selected iSNPs and their proxies (r^2^ ≥ 0.8, 1000 Genomes African population), identifying 148 SNPs. After removing 49 duplicates within LD blocks, 99 unique SNPs remained. Supplementary Table S3 provides regulatory information for all 99 SNPs with functional annotations, including histone marks, DNase hypersensitivity peaks, eQTL, and possible influence on protein binding and altered regulatory motifs from HaploReg analyses. After removing all duplicate variants, the remaining 99 SNPs were further investigated for their regulatory potential using RegulomeDB [35,36], FORGEdb [37], and the rSNPBase v3.0 [38] software packages to prioritize the most relevant regulatory SNPs (rSNPs). Supplementary Tables S4 and S5 describe the classification system corresponding to RegulomeDB and FORGEdb scores, respectively. Note that the RegulomeDB score of 1a–1f or 2 corresponds to a high potential for regulatory function. On the other hand, scores 8–10 in FORGEdb imply high regulatory potential, and rSNP is annotated as yes or no in rSNPBase. Supplementary Table S6 provides detailed information on the potential regulatory function of the SNPs in rSNPBase. The detailed results of RegulomeDB, FORGEdb, and rSNP are given in Supplementary Table S7, and the last column indicates whether the SNP is an rSNP from rSNPBase. Note that one SNP, rs11891071 (*ALK*), was not found in RegulomeDB or FORGEdb; therefore, only 98 SNP results are provided in Supplementary Tables S6 and S7.

The SNP prioritization process focused on identifying variants with the strongest regulatory evidence. Our SNP prioritization scheme involved (1) prioritizing the SNPs with a RegulomeDB score ≤ 2 (strong potential for regulatory consequence); and (2) selecting the SNPs if they had high RegulomeDB scores (1a–1f or 2a–2b), FORGEdb scores ≥ 8, and were annotated as regulatory variants in rSNPBase.

In the following Table 1, we provide SNPs with RegulomeDB scores of 1 or 2, indicating strong evidence of regulatory potential, along with their corresponding FORGEdb scores and regulatory SNP (rSNP) annotations from rSNPBase (yes/no). A RegulomeDB score ranging from 1a to 1f suggests that the SNP is likely to influence transcription factor (TF) binding and is linked to gene expression of a target gene, whereas a score of 2 indicates a likely effect on TF binding alone. In total, six SNPs were classified as 1b, two SNPs as 1d, 34 SNPs as 1f, one SNP as 2a, and one SNP as 2b in RegulomeDB.

The subsequent step involved prioritizing SNPs from Table 1 based on the following criteria: high RegulomeDB scores (1a–1f or 2a–2b), FORGEdb scores ≥ 8, and annotation as regulatory variants in rSNPBase. Applying these thresholds, we identified 7 candidate regulatory SNPs. Notably, rs1669707 exhibited strong linkage disequilibrium (r^2^ = 0.92) with the index SNP (iSNP) rs1669691. Table 2 presents HaploReg-derived functional annotations for the selected SNPs, including evidence of histone modifications, overlap with DNase hypersensitivity sites, expression quantitative trait loci (eQTL) associations, and potential effects on protein binding and regulatory motif disruption.

**Figure 2:**
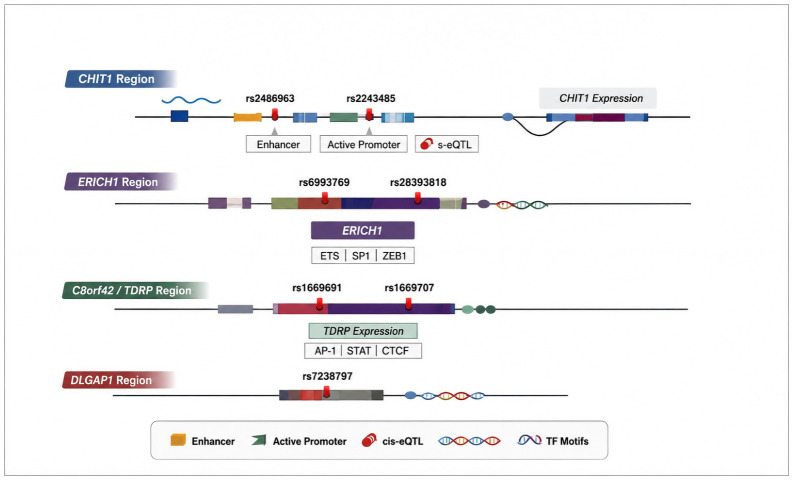
Regulatory variants implicated in *Chlamydia trachomatis* reinfection.

Supplementary Tables S8–S10 provide a detailed description of 7 SNPs overlapping with regulatory chromatin states from DNase I hypersensitivity sites (DHS) and histone marks from ChIP-Seq in several tissue/cell types, hits from selected eQTL studies, and overlapping regulatory motifs, respectively, from HaploReg. We summarized the results for the selected seven SNPs in Figure 2.

HaploReg v4.2 annotation demonstrated that rs2244385 and rs2486963 are intronic variants within *CHIT1* that localize to regulatory regions enriched for enhancer (H3K4me1, H3K27ac) and promoter (H3K4me3, H3K9ac) chromatin states across multiple immune and epithelial cell types (Supplementary Table S8). Both variants exhibit significant cis-eQTL associations with *CHIT1* expression in whole blood and other tissues, supporting their role in transcriptional regulation (Supplementary Table S9). Furthermore, these variants alter multiple transcription factor binding motifs. For example, rs2486963 disrupts binding motifs for transcription factors involved in immune regulation and chromatin architecture, including AhR, CTCF, and HEY1, and rs2244385 alters transcription factor binding motifs involved in epithelial and immune regulation, including AP-2 (epithelial differentiation and inflammatory regulation) [39] and BCL (immune cell survival and activation) [40], suggesting potential effects on chromatin architecture and immune signaling pathways (Supplementary Table S10). *CHIT1* encodes chitotriosidase, a macrophage- and neutrophil-derived enzyme induced during innate immune activation and infection [41,42,43,44]. Given evidence that host susceptibility to *Ct* is shaped by regulatory variation affecting myeloid inflammatory responses [45,46,47], rs2486963 and rs2244385 represent a plausible functional locus contributing to inter-individual differences in immune tone, bacterial persistence, and inflammatory pathology. Collectively, these findings support a regulatory mechanism whereby *CHIT1*-associated variants modulate host immune responses, thereby influencing susceptibility to *Ct* reinfection.

The rs1669691 and rs1669707 are in LD and are noncoding variants located upstream of the *TDRP* gene that map to regulatory regions enriched for both promoter (H3K4me3, H3K9ac) and enhancer (H3K4me1, H3K27ac) chromatin states across diverse cell types, including immune, epithelial, and mesenchymal lineages (Supplementary Table S8). Both variants exhibit significant cis-eQTL associations with *TDRP* expression in fibroblast and adipose tissues, indicating a role in transcriptional regulation (Supplementary Table S9). Furthermore, these variants alter multiple transcription factor binding motifs, including CTCF, SP1, AP-1, and STAT, suggesting potential effects on inflammatory signaling and chromatin organization (Supplementary Table S10) [48,49]. The *TDRP* (Testis Development–Related Protein) gene encodes a protein primarily involved in reproductive biology, particularly spermatogenesis. Although the biological role of *TDRP* is not well characterized outside reproductive biology, it encodes a nuclear and cytoplasmic protein primarily implicated in spermatogenesis and sperm function. The role of *TDRP* in *Ct* infection or reinfection in women remains unclear; however, it is expressed across the female reproductive tract, with the highest median transcripts per million (TPM) levels in the ovary, followed by the fallopian tube, endocervix, uterus, vagina, and ectocervix [50]. Note that fibroblasts play a critical role in shaping epithelial immunity and inflammatory tone during *Ct* infection [51,52], suggesting that regulatory variation at rs1669691 and rs1669707 may influence host transcriptional responses that determine bacterial persistence or pathology. Taken together, these observations suggest that *TDRP*-associated regulatory variation may contribute to host susceptibility by modulating tissue-specific inflammatory and epithelial responses during *Ct* reinfection.

The rs28393818 is an intronic variant within *ERICH1*, and rs6993769 is proximal to *ERICH1*. The exact biological and molecular function of the *ERICH1* (Glutamate Rich 1) gene remains largely uncharacterized. Both variants map to regulatory regions enriched for enhancer (H3K27ac) and, to a lesser extent, promoter (H3K4me3) chromatin states across immune and epithelial cell types (Supplementary Table S8). Both variants exhibit cell-type-specific regulatory activity in monocytes, fibroblasts, and keratinocytes, implicating roles in host defense and epithelial barrier function (Supplementary Table S8). Notably, rs6993769 demonstrates strong eQTL associations with *ERICH1* expression in whole blood (*p*-value = 1.02 × 10^−162^) and other tissues, supporting its role in transcriptional regulation (Supplementary Table S9). Additionally, these variants disrupt multiple transcription factor binding motifs, including IRF, ETS, SP1, and ZEB1, suggesting potential effects on interferon signaling, immune activation, and epithelial remodeling (Supplementary Table S10). Collectively, these findings indicate that regulatory variation at these loci may influence host susceptibility to *Ct* reinfection through modulation of gene expression and immune–epithelial interactions.

rs7238797 is an intronic variant within *DLGAP1* that overlaps DNase-accessible, enhancer- and promoter-flanking chromatin across mesenchymal, fibroblast, melanocyte cell types, and cervical epithelial cells relevant to *Ct* reinfection (Supplementary Table S8). HaploReg functional annotation indicates binding of transcriptional repressors such as NRSF/REST at this locus and allele-dependent disruption of multiple regulatory motifs involved in chromatin remodeling and immune regulation (Table 2 and Supplementary Table S10). Also, allele-dependent disruption of transcription-factor binding motifs, including BCL, SPIB, and the Sin3A/K-20, plays a central role in immune activation and transcriptional control [53,54,55]. Given the reliance of *Ct* on host cell signaling, transcriptional state, and intracellular niche control, rs7238797 represents a plausible regulatory locus contributing to inter-individual variability in infection persistence and disease outcome.

*Investigation of Ct reinfection genes targeted by microRNAs (miRNAs)*. Our prior *Ct* reinfection GWAS identified several genes, using chromatin interaction (CI) and eQTL analyses, including *CHIT1*, *ADORA1*, and *CHI3L1* (chromosome 1); *TDRP*, *FBXO25*, and *SULF1* (chromosome 8); and *SOCS6* (chromosome 18) [3], with *ALK* (chromosome 2) as the only non-CI/eQTL target. We used data mining to identify miRNAs targeting these genes. Table 3 provides miRNAs implicated in *Ct* infection [56].

The miRNA gene interaction landscape suggests that susceptibility to *Ct* reinfection is driven by layered post-transcriptional regulation. Notably, *SOCS6* and *SULF1* show extensive miRNAs targeting (miR-232 and miR-249, respectively), including *Ct*-associated miRNAs such as miR-16 [58,59,60], miR-155 [61,62,63], miR-21 [64], and miR-519d [65]. In contrast, *CHIT1* shows no annotated *Ct*-related miRNAs, suggesting a predominantly DNA-level regulatory mechanism. Recurrent miRNAs (e.g., miR-24 [60,66] and miR-16 [58,59,60]) target multiple genes, indicating coordinated regulation. Overall, these findings support a model in which reinfection risk reflects interactions among regulatory variants, transcriptional control, and miRNA networks rather than single-gene effects.

*CNV Analyses*. The genotyping data quality control and copy number determination analysis pipeline for CNV analysis is provided in Supplementary Figure S1. A total of 285 African American women passed SNP-level and sample-level quality control (QC) for CNV analysis. Among them, 60 cases experienced *Ct* reinfection, while 225 women served as controls. Initial CNV calling using post-QC intensity data identified 28,114 CNVs, encompassing 427,313 SNPs within CNV intervals. After removal of samples that failed intensity-based QC thresholds, 19,849 CNVs remained, with 154,546 SNPs excluded during sample-level QC (Supplementary Table S11). Applying CNV-level size and SNP-count thresholds further reduced the dataset. A total of 13,604 CNVs were removed across all filters, including CNVs smaller than 10 kb, larger than 1 Mb, or supported by ≤10 SNPs. After filtering, 6245 CNVs remained for merging. Merging adjacent CNVs reduced the number of events by 470, yielding a final high-confidence CNV set of 5775 CNVs for downstream analyses (Supplementary Table S11).

The distribution of CNV counts between cases and controls was similar. Cases carried a total of 1725 CNVs (mean 28.75 CNVs per person; median 8), while controls carried 4050 CNVs (mean 18 CNVs per person; median 8). The median CNV event length was 34,067 bp among cases and 31,920 bp among controls. Deletions accounted for 40.1% of CNVs in cases and 65.9% in controls, while duplications comprised 59.9% of CNVs in cases and 34.1% in controls (Supplementary Table S12). CNV sizes were consistent across groups. In cases, the median deletion length was 30,466 bp (interquartile range [IQR]: 19,446–57,827), and the median duplication length was 36,924 bp (IQR: 21,597–70,654). In controls, the median deletion and duplication lengths were 30,478 bp (IQR: 19,651–52,080) and 35,817 bp (IQR: 20,536–74,893), respectively. Call confidence values were also similar between groups. Deletions showed mean confidence scores of 52.45 in cases and 54.96 in controls, while duplications had mean confidence values of 46.68 and 65.25, respectively (Supplementary Table S12). Event size distributions were comparable across reinfection groups. Nearly all deletions were between 10 and 100 kb (89.0% in cases and 89.4% in controls), and most duplications also fell within this size range (84.1% in cases and 82.3% in controls). Only a small fraction of events exceeded 1 Mb (less than 1% in all groups) (Supplementary Table S12).

In gene-level analyses, nominal associations were identified at several biologically relevant loci (Supplementary Table S13). To clarify the main gene-level CNV findings, we summarized the prioritized nominal association signals in Table 4, while the complete gene-body CNV association results are provided in Supplementary Table S13.

Exonic deletions in *ATAD3A* (ATPase family AAA domain-containing protein 3A), *CARD14* (Caspase Recruitment Domain Family Member 14), and *TMEM240* (Transmembrane Protein 240) were nominally enriched in cases (OR = 8.78, *p* = 0.020), as were exonic duplications in *ZNF140* (OR = 5.48, *p* = 0.045). Intronic CNVs in *FAM166A*, *SSU72*, *SNORA1*, and *SNORA63* also showed nominal associations (*p* < 0.05). These associations did not remain significant after correction for genome-wide gene-level multiple testing and should therefore be interpreted as exploratory signals requiring replication. To assess the functional relevance of these findings, we examined tissue-specific expression using GTEx (Supplementary Figures S2–S4). *ATAD3A* is a nuclear-encoded mitochondrial membrane protein essential for mitochondrial dynamics, nucleoid organization, cholesterol metabolism, and protein translation. Deletions in the *ATAD3A* gene can drastically impair mitochondrial function. Exonic deletions in the *CARD14* gene generally lead to two contrasting outcomes depending on whether they cause a gain-of-function or loss-of-function at the cellular level [67]. *CARD14*, a scaffold protein involved in NF-κB–mediated inflammation [68], shows high expression in the ectocervix and skin (Supplementary Figure S2), using GTEx [69]. *ZNF140* encodes a Kruppel-associated box (KRAB) containing C2H2 zinc finger protein that acts as a sequence-specific transcriptional repressor. Because its primary role involves binding to specific DNA sequences to regulate gene expression, exonic duplications typically trigger distinct functional and physiological consequences. *ZNF140* exhibits elevated expression across female reproductive tissues, including the ovary, fallopian tube, uterus, and endo and ectocervix (Supplementary Figure S3), while *ATAD3A* is highly expressed in lymphocytes, fibroblasts, and the fallopian tube (Supplementary Figure S4). Although these associations did not remain significant after genome-wide multiple-testing correction, the convergence of nominal CNV signals with tissue-specific expression in STI-relevant tissues highlights *ATAD3A*, *CARD14*, and *ZNF140* as biologically plausible candidates for replication rather than confirmed susceptibility loci (Supplementary Table S13).

## 3. Discussion

This study provides integrative evidence that host susceptibility to urogenital *Ct* reinfection is primarily driven by noncoding regulatory variation rather than protein-altering mutations. By combining GWAS signals, functional annotations, miRNA targeting, and CNV profiling, we define a multilayered regulatory architecture underlying *Ct* reinfection risk.

A central finding is the prioritization of regulatory variants at key loci, particularly *CHIT1*, where intronic variants (e.g., rs2486963, rs2244385) show strong evidence of functional relevance, including active chromatin states, transcription factor binding disruption, and cis-eQTL effects in immune tissues. Functionally, *CHIT1* encodes chitotriosidase, a macrophage- and neutrophil-derived enzyme involved in innate immune activation and inflammatory responses [41,42,43,44]. Given the role of *CHIT1* in macrophage- and neutrophil-mediated innate immunity, regulatory perturbations at this locus may influence inflammatory responses, pathogen clearance, and susceptibility to reinfection [45,46,47]. These findings are consistent with emerging evidence that host susceptibility to *Ct* infection is shaped by regulatory variation affecting immune cell differentiation and macrophage polarization [45,46].

Additional loci, including variants upstream of *TDRP* (rs1669691, rs1669707) and near *ERICH1* (rs6993769, rs28393818), further support a model in which the transcriptional regulation across epithelial and stromal cell compartments contributes to host susceptibility. Disruption of transcription factor motifs such as AP-1, STAT, and ETS families highlights inflammatory signaling pathways [48,49,70]. Notably, fibroblasts and epithelial cells—key targets of *Ct* infection—emerge as important regulatory contexts that may influence intracellular niche permissiveness, immune signaling, and tissue repair [51,52,71].

miRNA analyses further support layered gene regulation, with genes such as *SOCS6* and *SULF1* targeted by multiple miRNAs, implicated in *Ct* infection (e.g., miR-16, miR-155, miR-21, miR-519d) [58,59,60,61,62,63,64,65]. These genes play important roles in cytokine signaling and extracellular matrix remodeling, suggesting that miRNA-mediated regulation may fine-tune inflammatory amplitude and resolution during repeated infections [56]. In contrast, *CHIT1* showed no known *Ct*-associated miRNA interactions, indicating that its effect may be driven primarily by DNA-level regulatory variation. The presence of shared miRNAs (e.g., miR-16 [58,59,60] and miR-24 [60,66]) targeting multiple genes suggests coordinated regulatory networks integrating transcriptional and post-transcriptional control [31,32].

CNV analysis provides additional evidence for structural variation as a complementary mechanism, with nominal associations identified at loci such as *CARD14* and *ATAD3A*, both of which are relevant to immune signaling and host–pathogen interactions. Although these findings require replication, their biological plausibility supports further investigation. These findings are consistent with prior literature demonstrating that CNVs can modulate host susceptibility to infectious diseases through gene-dosage effects and alteration of immune gene expression [4,29].

Collectively, these results support a model in which susceptibility to *Ct* reinfection arises from the interplay of regulatory SNPs, miRNA networks, and structural variants. These mechanisms converge on pathways governing immune activation, epithelial barrier function, inflammatory signaling, and tissue remodeling, emphasizing the central role of regulatory variation in shaping host responses to reinfection. This multi-layered regulatory architecture is consistent with broader observations in complex infectious diseases, where noncoding variation and regulatory networks play dominant roles in shaping host response [29].

### Limitations and Future Directions

This study has several limitations. It focused on African Americans, as the population from which patients were enrolled was mostly African Americans, and on women due to the disproportionate impact of *Ct* on women (causing perinatal and reproductive morbidity). There is also a lack of publicly available *Ct* reinfection datasets, limiting analyses in other populations and potentially affecting generalizability. We did not examine miRNA-targeted SNPs (e.g., 3′UTR variants affecting miRNA binding), instead focusing on genes targeted by *Ct*-associated miRNAs; this will be addressed in future work. Also, the modest CNV sample size reduced power to detect gene-level associations, and functional validation of regulatory variants was beyond the scope of this study. Additionally, regulatory annotations were derived from reference datasets rather than infection-specific contexts. Future studies integrating infection-state multi-omics, CRISPR-based validation, and replication in independent and diverse populations will be essential to confirm causal mechanisms and improve generalizability.

## 4. Materials and Methods

*Index SNPs Selection*. We selected 13 fine-mapping SNPs with Posterior Probability (PP) greater than or equal to 0.20, and 19 significant independent SNPs from recently published *Ct* reinfection GWAS with *p*-value < 1.0 × 10^−5^ [3]. We used 32 SNPs to investigate their functional regulatory potential.

*Prioritization of Regulatory Regions*. We used the functional implication of non-coding association signals using publicly available online resources, HaploReg v4.2 [34], RegulomeDB v2.0 [35], FORGEdb 2.0 [37], and rSNPBase v3.0 [38].

HaploReg provides annotations of noncoding SNPs within haplotype blocks by selecting a linkage disequilibrium (LD) threshold and using the 1000 Genomes reference populations. HaploReg provides SNPs in LD with query GWAS SNPs and their frequency in four super-populations from the 1000 Genomes Project. It uses ENCODE to identify regulatory protein binding, chromatin structure (cell types with DNase hypersensitivity), the chromatin state of the region (which can predict an enhancer or promoter), and putative transcription factor binding motifs altered by the variant.

The RegulomeDB scores for SNPs range from 1a to 7, providing potential for regulatory function, with lower scores indicating an increased likelihood of having a regulatory function. The RegulomeDB scores are based on information from expression quantitative trait loci (eQTLs) and chromatin marks.

In addition, we used FORGEdb scores to predict the GWAS SNPs’ regulatory relevance. In contrast to the RegulomeDB score, FORGEdb scores range between 0 and 10, with higher scores indicating an increased likelihood of having a regulatory function. In particular, FORGEdb scores are computed from the sum of the presence/absence of independent lines of regulatory evidence, including the eQTL (2 points), activity-by-contact (ABC) contacts (2 points), transcription factor (TF) motifs (1 point), contextual analysis of TF occupancy (CATO) score (1 point), DNase I hotspot (2 points), and histone mark ChIP-seq broad Peak (2 points). For example, a score of 10 or 9 suggests strong evidence for functional regulatory relevance compared to 0 or 1, indicating none or low evidence of regulatory functional relevance.

*Selection of Genes associated with Ct reinfection targeted by potential microRNAs (miRNAs)*. In the GWAS study in AA women, we identified multiple novel genes associated with *Ct* reinfection, including *CHIT1*, *CHI3L1*, *ADORA1*, *ALK*, *FBXO25*, *LINC01592*, *SULF1*, *SOCS6*, *ERICH1*, and *TDRP*, which are involved in the immune response due to infection [3]. We first obtained a list of miRNAs targeting the gene of interest from the GeneCards human gene database [72], which integrates data from miRTarBase [57]. Next, we cross-referenced these targets against the *Ct*-associated miRNAs identified in Table 1 of Meewes et al. to assess overlap with miRNAs targeting the identified genes [56].

### Copy Number Variations Analysis

*Study Sample*. The *Ct* immunogenetics study cohort has been described in detail in prior publications [3,73,74]. Briefly, the study enrolled females aged 16 years and older who presented to the Jefferson County Department of Health (JCDH) STD Clinic in Birmingham, Alabama, for treatment following a positive *Ct* nucleic acid amplification test (NAAT). All participants provided written informed consent before enrollment. Follow-up visits were scheduled for 3 and 6 months, during which participants completed additional interviews and provided repeat specimens. Reinfection was determined using repeat *Ct* NAAT testing. Exclusion criteria included pregnancy, prior hysterectomy, coinfection with HIV, syphilis, or gonorrhea at screening, immunosuppression, or antibiotic use with anti-chlamydial activity within the preceding 30 days.

*Genotype, Quality Control, and CNV Calling*. We conducted a CNV analysis using genome-wide SNP intensity data. Samples were genotyped using the Illumina Global Diversity Array v1.0, which contains 1,882,942 variants. Following variant-level quality control, which excluded monomorphic variants, insertions/deletions, variants with more than 5% missingness, non-autosomal variants, low minor allele frequency variants, and low-quality or duplicate samples, the dataset comprised 285 individuals and 1,074,163 variants (Supplementary Figure S1).

Allele-specific fluorescence intensities for all SNP probes were extracted and normalized using Illumina GenomeStudio software version 2.0.4. For each probe, GenomeStudio calculates the log R ratio (LRR), defined as the log_2_ ratio of the observed normalized probe intensity to the expected intensity, and the B allele frequency (BAF), which represents the allelic proportion [75]. In diploid regions without copy-number variation, LRR values center near zero, and BAF values cluster at 0, 0.5, or 1, corresponding to genotypes AA, AB, and BB. Deviations from these expected patterns were used to infer copy number changes.

CNV calling was performed on all individuals with available post-QC LRR and BAF values using PennCNV [76,77,78], which employs a hidden Markov model that incorporates LRR, BAF, population allele frequency, and inter-marker distance [76,77,78]. Sample-level CNV quality control was subsequently applied, excluding individuals with an LRR standard deviation greater than 0.25 or a total CNV count exceeding 1000 events.

To minimize false positive calls and establish a high-confidence dataset, we applied a rigorous CNV-level filtering strategy informed by the methodology described by Xiao et al. [79] CNVs smaller than 10 kb, larger than 1 Mb, or supported by ≤10 SNPs were removed. These filtering criteria reduced the dataset to 6245 CNVs. Finally, to address the fragmentation of single events, adjacent CNVs separated by a gap of less than 20% of their combined length were merged. This pipeline resulted in a final high-confidence CNV set of 5775 events used for downstream analyses described in Section 2 (Supplementary Table S11).

Gene-level CNV association testing was performed using hg38/GRCh38 coordinates and UCSC knownGene annotations implemented in ParseCNV2 [80]. Deletions and duplications were analyzed separately. For each gene, individuals were classified as CNV carriers if they had at least one high-confidence CNV overlapping the annotated gene body, defined as the transcript span of the UCSC knownGene entry, including both exons and introns. Exonic CNV carriers were defined as individuals whose CNV overlapped an annotated exon, whereas intronic-only carriers were defined as individuals whose CNV overlapped the gene body but did not overlap annotated exons. Gene-level case–control enrichment was evaluated by comparing carrier counts between *Ct* reinfection cases and controls for each CNV type. These results are summarized in Supplementary Table S13, where exonic gene-body hits are listed first, followed by additional intronic-only gene-body hits.

## 5. Conclusions

In summary, this study demonstrates that host susceptibility to urogenital *Ct* reinfection is predominantly shaped by non-coding regulatory genetic variation rather than protein-altering mutations. Integrative analyses of GWAS signals, regulatory annotations, miRNA targeting, and copy number variation highlight transcriptional regulation of immune and epithelial pathways, particularly at loci such as *CHIT1*, *TDRP*, *ERICH1*, *SULF1*, and *SOCS6*, as central contributors to reinfection risk. Together, these findings support a model in which layered regulatory mechanisms modulate immune response, inflammatory resolution, and tissue responses, providing a framework for future functional studies and the development of genetically informed strategies for prevention and risk stratification of *Ct* reinfection.

## Figures and Tables

**Table 1:** RegulomeDB scores (categories of 1 or 2) to evaluate the regulatory potential of *Chlamydia trachomatis* reinfection index SNPs (iSNPs) and SNPs in high linkage disequilibrium (LD) of ≥ 0.8 with these iSNPs, and the corresponding RegulomeDB score, FORGEdb score, and indication of regulatory SNP (rSNP) by rSNPBase.

iSNP	Chr	pos (hg38)	LD (r^2^)	SNP	Genes	RegulomeDB Score	FORGEdb Score	rSNP from rSNPBase
rs2486961	1	203220506	0.94	rs2486959	*CHIT1*	1f	6	Yes
	1	203222134	0.82	rs2244385	*CHIT1*	1f	8	Yes
	1	203222776	1	rs2486961	*CHIT1*	1f	6	Yes
	1	203223320	0.82	rs3216011	*CHIT1*	1f	2	Yes
rs1417150	1	203219564	0.84	rs2486958	*CHIT1*	1f	6	Yes
	1	203220360	0.84	rs1002485	*CHIT1*	1f	6	Yes
	1	203220424	0.84	rs1556854	*CHIT1*	1f	6	Yes
	1	203222792	0.84	rs2486962	*CHIT1*	1f	6	Yes
	1	203224880	0.87	rs2486963	*CHIT1*	1f	8	Yes
	1	203227629	1	rs1417150	*CHIT1*	1f	6	Yes
rs1669691	8	540864	0.83	rs11996757	*TDRP*	1f	7	No
	8	546505	1	rs1669691	1.2kb 5′ of *TDRP*	1f	10	Yes
	8	547247	0.92	rs1669707	1.9kb 5′ of *TDRP*	1f	8	Yes
rs1703937	8	656452	1	rs1703937	7.7kb 3′ of *ERICH1*	1d	7	No
rs4735900	8	655791	0.92	rs144650377	8.4kb 3′ of *ERICH1*	1b	2	No
	8	656117	0.83	rs1669681	8.1kb 3′ of *ERICH1*	1b	7	No
	8	656200	0.94	rs6996811	8kb 3′ of *ERICH1*	1f	8	No
	8	657161	1	rs4735900	7kb 3′ of *ERICH1*	1f	7	Yes
	8	658018	0.81	rs6993769	6.2kb 3′ of *ERICH1*	1f	8	Yes
	8	658386	0.86	rs28393818	5.8kb 3′ of *ERICH1*	1b	8	Yes
rs7238797	18	3554526	1	rs7238797	*DLGAP1*	2a	8	Yes
rs28505079	18	70469178	1	rs28580582	139kb 3′ of *SOCS6*	1f	7	No
	18	70469288	1	rs28505079	139kb 3′ of *SOCS6*	1b	7	No
	18	70470013	1	rs56756677	140kb 3′ of *SOCS6*	1f	8	No
	18	70471721	1	rs28459797	142kb 3′ of *SOCS6*	1f	7	No
	18	70471844	1	rs28759291	142kb 3′ of *SOCS6*	1f	6	No
	18	70471997	1	rs17082653	142kb 3′ of *SOCS6*	1f	7	No
	18	70472708	0.99	rs9953912	143kb 3′ of *SOCS6*	1f	6	No
	18	70472757	0.98	rs9964993	143kb 3′ of *SOCS6*	1f	6	No
	18	70473290	1	rs58830422	143kb 3′ of *SOCS6*	1f	7	No
	18	70473428	0.97	rs9956900	143kb 3′ of *SOCS6*	1f	5	No
	18	70473593	0.88	rs9944770	143kb 3′ of *SOCS6*	1d	8	No
	18	70475478	0.86	rs8093705	145kb 3′ of *SOCS6*	1f	6	No
	18	70477745	0.84	rs60088432	148kb 3′ of *SOCS6*	1f	5	No
	18	70478074	0.86	rs7233902	148kb 3′ of *SOCS6*	1b	6	No
	18	70478307	0.85	rs7234494	148kb 3′ of *SOCS6*	1b	6	No
	18	70478459	0.85	rs7234834	148kb 3′ of *SOCS6*	1f	6	No
rs28373933	18	70469147	1	rs28373933	139kb 3′ of *SOCS6*	1f	8	No
	18	70475685	0.87	rs8092963	145kb 3′ of *SOCS6*	1f	6	No
	18	70477271	0.87	rs73459349	147kb 3′ of *SOCS6*	1f	7	No
	18	70477392	0.87	rs73459351	147kb 3′ of *SOCS6*	1f	6	No
	18	70478289	0.85	rs7234487	148kb 3′ of *SOCS6*	1f	4	No
rs9965095	18	70472869	1	rs9965095	143kb 3′ of *SOCS6*	1f	5	No
rs113862101	20	1799904	1	rs113862101	20kb 5′ of *LOC100289473*	2b	5	No

**Table 2:** Annotation of 7 regulatory *Chlamydia trachomatis* reinfection SNPs in HaploReg with RegulomeDB Scores of 1 or 2, FORGEdb score of at least 8, and the regulatory SNP is identified by the rSNPBase database.

chr.	pos (hg38)	LD (r^2^)	Variant	Ref/Alt	Genes	dbSNP Functional Annotation	Promoter/Enhancer Histone Marks	DNase	Proteins Bound	Motifs Altered	Selected eQTL Hits
Query iSNP: rs1417150 and variants with r^2^ ≥ 0.8											
1	203224880	0.87	rs2486963	A/G	*CHIT1*	intronic	Yes			9 altered motifs ^1^	Yes
Query iSNP: rs2486961 and variants with r^2^ ≥ 0.8											
1	203222134	0.82	rs2244385	C/G	*CHIT1*	intronic	Yes			AP-2, BCL	Yes
Query iSNP: rs1669691 and variants with r^2^ ≥ 0.8											
8	546505	1	rs1669691	C/G	1.2kb 5′ of *TDRP*	intergenic	Yes	Yes		13 altered motifs ^2^	Yes
8	547247	0.92	rs1669707	G/C	1.9kb 5′ of *TDRP*	intergenic	Yes	Yes		AP-1, Foxa, STAT	Yes
Query iSNP: rs4735900 and variants with r^2^ ≥ 0.8											
8	658018	0.81	rs6993769	C/T	6.2kb 3′ of *ERICH1*	intergenic				9 altered motifs ^3^	Yes
8	658386	0.86	rs28393818	G/C	5.8kb 3′ of *ERICH1*	intronic				12 altered motifs ^4^	No
Query iSNP: rs7238797 and variants with r^2^ ≥ 0.8											
18	3554526	1	rs7238797	T/C	*DLGAP1*	intronic	Yes	Yes	NANOG, NRSF	6 altered motifs ^5^	No

^1^ AP-4_2, AhR_1, CTCF_disc7, HEY1_disc2, LBP-1_2, Lmo2-complex_1, Nanog_disc3, Rad21_disc6, SREBP_known2. ^2^ AP-2_known2, CAC-binding-protein, CTCF_disc8, ERalpha-a_disc4, EWSR1-FLI1, MAZ, Pax-5_known3, Rad21_disc6, SP1_known1, STAT_disc7, TFII-I, WT1, Znf143_disc3. ^3^ CACD_2, DEC, EBF_disc2, HEN1_1, LXR_1, MAZR, Mxi1_known1, PLAG1, ZEB1_disc1. ^4^ BCL_disc1, BDP1_disc1, CACD_2, CCNT2_disc2, CTCF_disc8, ELF1_disc1, ELF1_disc2, Ets_disc2, Ets_known3, Ets_known5, Irf_disc4, SP1_disc3, SP1_known2, Sp4, Zfp281, Zfp740. ^5^ BCL_disc3, NRSF_disc2, NRSF_disc4, NRSF_known1, NRSF_known3, SPIB, Sin3Ak-20_disc1, Sin3Ak-20_disc3, TEF-1_2, TFII-I.

**Table 3:** Compilation of human and mouse microRNAs predicted to target genes implicated in *Chlamydia trachomatis* reinfection GWAS [3], based on annotations from the miRTarBase database [57], and *Chlamydia* infection microRNAs described in Meewes et al. [56].

Gene	Number of miRNAs Targeting the Gene	Known Chlamydia Associated miRNAs in Humans	Known Chlamydia Associated miRNAs in the Mouse
*ADORA1*	58	miR-16, miR-24	miR-15a, miR-16, miR-22, miR-24
*ALK*	2	None	miR-132-3p
*CHI3L1*	34	miR-24	miR-24, miR-214
*CHIT1*	25	None	None
*FBXO25*	39	None	miR-23b, miR-214
*SOCS6*	232	miR-16, miR-155, miR-519d, miR-559	miR-15a, miR-16, miR-21, miR-23b, miR-27a, miR-30c, miR-30e, miR-128, miR-155, miR-182, miR-183
*SULF1*	249	miR-449a, miR-449c, miR-519d	miR-19a, miR-105, miR-212, miR-214, miR-378b, miR-429
*TDRP*	4	miR-24-3p, miR-221-3p	None
*ERICH1*	7	None	None

**Table 4:** Summary of prioritized gene-level CNV association signals in *Chlamydia trachomatis* reinfection cases and controls.

Gene	CNV Type	CNV Overlap Category	Case Carriers	Control Carriers	OR [95% CI]	*p*-Value	Interpretation
*ATAD3A*	Deletion	Exonic	3/55 (5.45%)	1/198 (0.51%)	8.78 [1.41, 91.99]	0.020	Prioritized exonic deletion signal
*CARD14*	Deletion	Exonic	3/55 (5.45%)	1/198 (0.51%)	8.78 [1.41, 91.99]	0.020	NF-κB/inflammatory signaling candidate
*TMEM240*	Deletion	Exonic	3/55 (5.45%)	1/198 (0.51%)	8.78 [1.41, 91.99]	0.020	Prioritized exonic deletion signal
*ZNF140*	Duplication	Exonic	3/54 (5.56%)	2/203 (0.99%)	5.48 [1.04, 33.63]	0.045	Transcriptional regulation candidate
*SNORA1*	Duplication	Intronic-only gene-body	7/55 (12.73%)	9/216 (4.17%)	3.38 [1.20, 9.25]	0.023	Additional intronic-only gene-body signal
*SNORA63*	Duplication	Intronic-only gene-body	9/55 (16.36%)	14/216 (6.48%)	2.85 [1.15, 6.80]	0.025	Additional intronic-only gene-body signal

Note that CNV associations are nominal and did not remain significant after genome-wide gene-level multiple-testing correction. Deletions and duplications were analyzed separately. Detailed gene-body results are provided in Supplementary Table S13.

## Data Availability

The data presented in the study are deposited in the dbGaP repository, accession number phs004338.v1.p1.

## Supplementary Materials

The following supporting information can be downloaded at: https://www.mdpi.com/article/10.3390/ijms27125410/s1.
