# Supplementary material for: Exploration of Regulatory Elements, MicroRNAs, and Copy Number Variation in Urogenital Chlamydia Reinfection in African American Women
Source: Int J Mol Sci. 2026 Jun 16;27(12):5410. doi: 10.3390/ijms27125410 (PMC13300433; doi:10.3390/ijms27125410)
Supplement: Supplementary file 1 [file ijms-27-05410-s001.zip › ijms-4357461-supplementary.pdf]

Supplementary Table S1: Significant Fine-Mapping Single Nucleotide Polymorphisms (SNPs) for *Chlamydia Trachomatis* (*Ct*) reinfection with Posterior Probability greater than or equal to 0.20 identified in Tiwari *et al*. (2025).[1]

SNP rsID	Chr.	BP hg19	BP hg38	Gene	P-Value GWAS	Posterior Prob	Function
rs2486961	chr1	203191904	203222776	*CHIT1*	3.87E-06	0.788	Intronic
rs1417150	chr1	203196757	203227629	*CHIT1*	2.10E-02	0.632	Intronic
rs2486963	chr1	203194008	203224880	*CHIT1*	3.59E-02	0.201	Intronic
rs13390546	chr2	29946273	29723407	*ALK*	8.62E-03	0.814	intronic
rs111891071	chr2	29950711	29727845	*ALK*	2.13E-06	0.209	intronic
rs66953037	chr2	29950987	29728121	*ALK*	2.13E-06	0.209	intronic
rs1703937	chr8	606452	656452	*ERICH1*	1.33E-02	0.998	intronic
rs4735900	chr8	607161	657161	*ERICH1*	4.62E-02	0.587	intronic
rs6996811	chr8	606200	656200	*ERICH1*	4.68E-02	0.412	intronic
rs6998830	chr8	69843088	68930853	*LINC01592*	2.61E-05	1.000	ncRNA intronic
rs4737926	chr8	69850612	68938377	*LINC01592*	9.98E-05	0.998	ncRNA intronic
rs28373933	chr18	68136383	70469147	*LINC01910-GTSCR1*	7.49E-06	0.517	intergenic
rs9965095	chr18	68140105	70472869	*LINC01910-GTSCR2*	2.49E-03	0.261	intergenic

Supplementary Table S2: All independent Single Nucleotide Polymorphisms (SNPs) from *Chlamydia Trachomatis* (*Ct*) reinfection GWAS with P-value <1.0E-05.[1]

uniqeID	rsID	Chr.	Base Pair Position (hg19)	Base Pair Position (hg38)	Gene	Function	P-value Ct-reinfection GWAS
chr1:203191904:C:T	rs2486961	1	203191904	203222776	*CHIT1*	intronic	3.87E-06
chr2:29950711:C:CAG	rs111891071	2	29950711	29,727,845	*ALK*	intronic	2.13E-06
chr2:52414476:G:T	rs1922198	2	52414476	52,187,338	*LOC730100*	ncRNA Intronic	7.84E-06
chr5:11742434:C:G	rs10474933	5	11742434	11,742,322	*CTNND2*	intronic	8.08E-06
chr5:11742674:G:T	rs4702814	5	11742674	11,742,562	*CTNND2*	intronic	8.16E-06
chr5:116241425:A:C	rs1979248	5	116241425	116,905,729	*LINC02214-LINC00992*	intergenic	6.95E-06
chr5:174106526:A:G	rs17063846	5	174106526	174,679,523	*LINC01411-MSX2*	intergenic	9.89E-06
chr6:107228541:C:T	rs113237398	6	107228541	106,780,666	*LOC100422737*	ncRNA Intronic	3.26E-06
chr6:149199153:A:G	rs28530774	6	149199153	148,878,017	*UST*	intronic	2.63E-06
chr8:496505:C:G	rs1669691	8	496505	546,505	*TDRP*	upstream	3.64E-07
chr8:69843242:A:G	rs6999003	8	69843242	68,931,007	*LINC01592*	ncRNA Intronic	3.98E-06
chr8:69853641:C:G	rs66891172	8	69853641	68,941,406	*LINC01592*	ncRNA Intronic	3.45E-06
chr10:79522198:G:GTTTA	rs144396310	10	79522198	77,762,440	*KCNMA1-DLG5*	intergenic	8.14E-06
chr10:108435513:A:G	rs821932	10	108435513	106,675,755	*SORCS1*	intronic	6.29E-06
chr16:13212968:A:G	rs72784472	16	13212968	13,119,111	*SHISA9*	intronic	6.44E-06
chr18:3423019:A:G	rs73375993	18	3423019	3,423,021	*TGIF1*	intronic	6.27E-06
chr18:3554524:C:T	rs7238797	18	3554524	3,554,526	*DLGAP1*	intronic	5.11E-06
chr18:68136524:A:G	rs28505079	18	68136524	70,469,288	*LINC01910-GTSCR1*	intergenic	6.71E-06
chr20:1780550:C:T	rs113862101	20	1780550	1,799,904	*LOC100289473-SIRPA*	intergenic	7.12E-06

Supplementary Table S3: HaploReg v4.2 results for unique 99 SNPs, including index Single Nucleotide Polymorphisms (iSNPs) associated with *Chlamydia Trachomatis* (*Ct*) and proxy SNPs in linkage disequilibrium (LD) with $r^2 \geq 0.8$.[2]

chr.	pos (hg38)	LD (r^2)	variant	Ref	Alt	Promoter histone marks	Enhancer histone marks	DNAse	Proteins bound	Motifs changed	Selected eQTL hits	RefSeq genes	dbSNP func annot
Query SNP: rs2486961 and variants with r^2 >= 0.8													
1	203220506	0.94	rs2486959	A	G					Gfi1, TATA	2 hits	*CHIT1*	intronic
1	203222134	0.82	rs2244385	C	G		BLD			AP-2, BCL	2 hits	*CHIT1*	intronic
1	203222776	1	rs2486961	C	T		BLD			GZF1, RP58	2 hits	*CHIT1*	intronic
1	203223320	0.82	rs3216011	GC	G		ESDR, PANC, LIV			4 altered motifs		*CHIT1*	intronic
Query SNP: rs1417150 and variants with r^2 >= 0.8													
1	203219564	0.84	rs2486958	A	C		BLD			HNF4	2 hits	*CHIT1*	intronic
1	203220360	0.84	rs1002485	T	C					Foxa, Foxd3, Pbx-1	1 hit	*CHIT1*	intronic
1	203220424	0.84	rs1556854	T	C	SKIN				GATA	2 hits	*CHIT1*	intronic
1	203222792	0.84	rs2486962	G	A		BLD				2 hits	*CHIT1*	intronic
1	203224880	0.87	rs2486963	A	G	GI	BLD, MUS			9 altered motifs	2 hits	*CHIT1*	intronic
1	203227629	1	rs1417150	T	C		BLD			Irx, Pou2f2, Pou3f2	2 hits	*CHIT1*	intronic
Query SNP: rs1922198 and variants with r^2 >= 0.8													
2	52186796	0.88	rs112614653	A	ACA AT					4 altered motifs		1.2Mb 5' of *NRXN1*	intronic
2	52187338	1	rs1922198	T	G					Dobox4, Pou2f2		1.2Mb 5' of *NRXN1*	intronic
Query SNP: rs13390546 and variants with r^2 >= 0.8													
2	29723407	1	rs13390546	C	A		4 tissues	5 tissues				*ALK*	intronic
Query SNP: rs66953037 and variants with r^2 >= 0.8													
2	29707337	0.8	rs112880022	C	T					Hsf, Smad4		*ALK*	intronic
2	29707453	0.8	rs79445350	C	T							*ALK*	intronic

chr.	pos (hg38)	LD (r^2)	variant	Ref	Alt	Promoter histone marks	Enhancer histone marks	DNAse	Proteins bound	Motifs changed	Selected eQTL hits	RefSeq genes	dbSNP func annot
2	29709229	0.8	rs80328971	C	T				NFKB			*ALK*	intronic
2	29711878	0.82	rs13382715	T	C					E2F		*ALK*	intronic
2	29716578	0.8	rs74904880	C	T		ESDR, BLD, SKIN			GR, Zic		*ALK*	intronic
2	29718920	0.91	rs77791547	C	G	ESDR	7 tissues					*ALK*	intronic
2	29724583	0.83	rs10175802	C	G			BLD		Pax-5		*ALK*	intronic
2	29726905	0.98	rs17008540	T	C		ESDR, BRN			Zfp410		*ALK*	intronic
2	29727845	0.98	rs111891071	C	CAG		ESDR, BRN			Pou2f2, Sin3Ak-20		*ALK*	intronic
2	29728121	1	rs66953037	TA	T		ESDR, BRN			8 altered motifs		*ALK*	intronic
2	29729627	0.98	rs113164730	T	C	4 tissues	7 tissues	BLD,BLD,BRN		PU.1, Pax-4		*ALK*	intronic
2	29730914	0.93	rs10208306	A	G			IPSC,BLD	EBF1			*ALK*	intronic
2	29732910	0.82	rs112576297	T	TC					7 altered motifs		*ALK*	intronic
2	29734091	0.82	rs13421314	A	G		BRN	BRN,BRN		5 altered motifs		*ALK*	intronic
2	29734360	0.82	rs6735852	A	C			BRN		5 altered motifs		*ALK*	intronic
2	29735424	0.83	rs79232623	T	C					Cdx		*ALK*	intronic
2	29741232	0.82	rs10211451	T	A,G				MAFK			*ALK*	intronic
2	29741521	0.8	rs77158497	T	A					6 altered motifs		*ALK*	intronic
Query SNP: rs10474933 and variants with r^2 >= 0.8													
5	11742322	1	rs10474933	C	G					Cdx2		*CTNND2*	intronic
Query SNP: rs4702814 and variants with r^2 >= 0.8													
5	11742562	1	rs4702814	G	T		ESC			12 altered motifs		*CTNND2*	intronic

chr.	pos (hg38)	LD (r^2)	variant	Ref	Alt	Promoter histone marks	Enhancer histone marks	DNAse	Proteins bound	Motifs changed	Selected eQTL hits	RefSeq genes	dbSNP func annot
Query SNP: rs1979248 and variants with r^2 >= 0.8													
5	116905729	1	rs1979248	C	A		BLD			Arid5a		331kb 5' of *SEMA6A*	
Query SNP: rs17063846 and variants with r^2 >= 0.8													
5	174677709	0.94	rs116039677	G	A					Hbp1		47kb 5' of *MSX2*	
5	174679523	1	rs17063846	A	G							45kb 5' of *MSX2*	
Query SNP: rs113237398 and variants with r^2 >= 0.8													
6	106780666	1	rs113237398	C	T	PANC	LIV, GI			Nr2f2		*LOC100422737*	intronic
Query SNP: rs28530774 and variants with r^2 >= 0.8													
6	148878017	1	rs28530774	A	G			BRN,OVRY		4 altered motifs		*UST*	intronic
Query SNP: rs1669691 and variants with r^2 >= 0.8													
8	540864	0.83	rs11996757	G	A	ESC, IPSC	ESC, IPSC, LIV	ESDR,SKIN		Hdx	3 hits	*TDRP*	intronic
8	541098	0.83	rs7829447	C	T	IPSC	ESC, IPSC, LIV			12 altered motifs	3 hits	*TDRP*	intronic
8	546505	1	rs1669691	C	G	10 tissues	8 tissues	4 tissues		13 altered motifs	1 hit	1.2kb 5' of *TDRP*	
8	547247	0.92	rs1669707	G	C		4 tissues	7 tissues		AP-1, Foxa, STAT	2 hits	1.9kb 5' of *TDRP*	
Query SNP: rs6999003 and variants with r^2 >= 0.8													
8	68931007	1	rs6999003	G	A		LNG, MUS	MUS		TATA		*LOC100505718*	intronic
8	68934516	0.89	rs71517216	A	C					Isl2, PLZF, Pax-6		*LOC100505718*	intronic
8	68935511	0.92	rs60914690	CAG	C		SKIN	SKIN		13 altered motifs		*LOC100505718*	intronic
8	68936817	0.87	rs34207383	A	G					20 altered motifs		*LOC100505718*	intronic

chr.	pos (hg38)	LD (r^2)	variant	Ref	Alt	Promoter histone marks	Enhancer histone marks	DNAse	Proteins bound	Motifs changed	Selected eQTL hits	RefSeq genes	dbSNP func annot
8	68937426	0.93	rs147607144	AAAC	A					FAC1, Foxp1, RREB-1		*LOC100505718*	intronic
8	68938718	0.92	rs60283435	C	T					6 altered motifs		*LOC100505718*	intronic
Query SNP: rs66891172 and variants with r^2 >= 0.8													
8	68941406	1	rs66891172	C	G			MUS		5 altered motifs		*LOC100505718*	intronic
8	68943499	0.86	rs7001684	G	A					YY1		*LOC100505718*	intronic
Query SNP: rs1703937 and variants with r^2 >= 0.8													
8	656452	1	rs1703937	T	A					6 altered motifs	3 hits	7.7kb 3' of *ERICH1*	
Query SNP: rs4735900 and variants with r^2 >= 0.8													
8	655791	0.92	rs144650377	TTC	T					Hoxd10, Sox		8.4kb 3' of *ERICH1*	
8	656117	0.83	rs1669681	G	A			LNG		Ik-1, NF-kappaB	4 hits	8.1kb 3' of *ERICH1*	
8	656200	0.94	rs6996811	G	C					5 altered motifs	1 hit	8kb 3' of *ERICH1*	
8	657161	1	rs4735900	T	C				SETDB1	Nanog	2 hits	7kb 3' of *ERICH1*	
8	658018	0.81	rs6993769	C	T					9 altered motifs	1 hit	6.2kb 3' of *ERICH1*	
8	658386	0.86	rs28393818	G	C					12 altered motifs		5.8kb 3' of *ERICH1*	intronic
Query SNP: rs6998830 and variants with r^2 >= 0.8													
8	68930853	1	rs6998830	G	A, C, T							*LOC100505718*	intronic
Query SNP: rs4737926 and variants with r^2 >= 0.8													
8	68938377	1	rs4737926	C	T					4 altered motifs		*LOC100505718*	intronic
8	68940441	0.86	rs6472431	A	G	MUS	BRST, MUS	BRST, MUS, MUS		Pax-4		*LOC100505718*	intronic

chr.	pos (hg38)	LD (r^2)	variant	Ref	Alt	Promoter histone marks	Enhancer histone marks	DNAse	Proteins bound	Motifs changed	Selected eQTL hits	RefSeq genes	dbSNP func annot
8	68947358	0.9	rs4737928	C	T					Foxa, Gm397		*LOC100505718*	intronic
Query SNP: rs144396310 and variants with r^2 >= 0.8													
10	77762440	1	rs144396310	GTTTA	G					19 altered motifs		28kb 3' of *DLG5*	
Query SNP: rs821932 and variants with r^2 >= 0.8													
10	106675755	1	rs821932	A	G					AIRE, Maf		*SORCS1*	intronic
10	106724757	0.83	rs1358874	G	A					NF-kappaB, Nkx2		*SORCS1*	intronic
16	13119111	1	rs72784472	G	A			ESDR		HEN1, Sp4		*SHISA9*	intronic
Query SNP: rs73375993 and variants with r^2 >= 0.8													
18	3423021	1	rs73375993	A	G		LNG, CRVX	CRVX	CEBPB	4 altered motifs		*TGIF1*	intronic
Query SNP: rs7238797 and variants with r^2 >= 0.8													
18	3554526	1	rs7238797	T	C	ESC, ESDR, IPSC	5 tissues	19 tissues	NANOG, NRSF	6 altered motifs		*DLGAP1*	intronic
Query SNP: rs28505079 and variants with r^2 >= 0.8													
18	70469178	1	rs28580582	G	A		BRST, BRN, SKIN	SKIN		BCL, Zfx		139kb 3' of *SOCS6*	
18	70469288	1	rs28505079	G	A		BRST, BRN, SKIN	BLD, SKIN		7 altered motifs		139kb 3' of *SOCS6*	
18	70470013	1	rs56756677	A	G	BRN	6 tissues	12 tissues	NRSF, P300, YY1	BCL, Pax-5		140kb 3' of *SOCS6*	
18	70471165	1	rs73459320	T	C		BRN			7 altered motifs		141kb 3' of *SOCS6*	
18	70471238	1	rs111352946	T	C		BRN					141kb 3' of *SOCS6*	
18	70471721	1	rs28459797	T	C					6 altered motifs		142kb 3' of *SOCS6*	

chr.	pos (hg38)	LD (r^2)	variant	Ref	Alt	Promoter histone marks	Enhancer histone marks	DNAse	Proteins bound	Motifs changed	Selected eQTL hits	RefSeq genes	dbSNP func annot
18	70471844	1	rs28759291	G	A					Gm397		142kb 3' of *SOCS6*	
18	70471997	1	rs17082653	C	T					Myf, RORalpha1, RP58		142kb 3' of *SOCS6*	
18	70472708	0.99	rs9953912	T	A					Pax-2, Pou1f1		143kb 3' of *SOCS6*	
18	70472757	0.98	rs9964993	G	A							143kb 3' of *SOCS6*	
18	70473290	1	rs58830422	A	T					4 altered motifs		143kb 3' of *SOCS6*	
18	70473428	0.97	rs9956900	T	G		BLD			Foxa, Pax-8, YY1		143kb 3' of *SOCS6*	
18	70473593	0.88	rs9944770	A	G		BLD		GATA2	Ets, Pax-4, STAT		143kb 3' of *SOCS6*	
18	70474677	0.84	rs7232674	G	A					CIZ, PLZF, SREBP		144kb 3' of *SOCS6*	
18	70475211	0.86	rs8092458	G	A					Foxp1, Hoxa5		145kb 3' of *SOCS6*	
18	70475478	0.86	rs8093705	T	C					AP-2, Foxj1		145kb 3' of *SOCS6*	
18	70476552	0.86	rs9954746	G	A					AIRE, Sox, Zfp410		146kb 3' of *SOCS6*	
18	70476930	0.8	rs35794277	C	T					HNF4		147kb 3' of *SOCS6*	
18	70477745	0.84	rs60088432	T	C			SKIN		4 altered motifs		148kb 3' of *SOCS6*	
18	70478074	0.86	rs7233902	C	T					4 altered motifs		148kb 3' of *SOCS6*	
18	70478307	0.85	rs7234494	G	C					5 altered motifs		148kb 3' of *SOCS6*	
18	70478459	0.85	rs7234834	G	A					BDP1, HEN1, Zfp691		148kb 3' of *SOCS6*	
18	70479803	0.82	rs7240698	G	C					GLI		150kb 3' of *SOCS6*	
Query SNP: rs28373933 and variants with r^2 >= 0.8													

chr.	pos (hg38)	LD (r^2)	variant	Ref	Alt	Promoter histone marks	Enhancer histone marks	DNAse	Proteins bound	Motifs changed	Selected eQTL hits	RefSeq genes	dbSNP func annot
18	70469147	1	rs28373933	C	T	BRN	BRST, BRN, SKIN	SKIN		6 altered motifs		139kb 3' of *SOCS6*	
18	70475178	0.87	rs8092443	G	A					DMRT2, TCF4		145kb 3' of *SOCS6*	
18	70475685	0.87	rs8092963	C	T					Nkx2, Pbx-1, Pbx3		145kb 3' of *SOCS6*	
18	70477271	0.87	rs73459349	T	C					4 altered motifs		147kb 3' of *SOCS6*	
18	70477392	0.87	rs73459351	T	C					HDAC2, TATA		147kb 3' of *SOCS6*	
18	70478289	0.85	rs7234487	G	A					STAT		148kb 3' of *SOCS6*	
Query SNP: rs9965095 and variants with r^2 >= 0.8													
18	70472869	1	rs9965095	G	A			OVRY		Foxd3, Sox, Zec		143kb 3' of *SOCS6*	
Query SNP: rs113862101 and variants with r^2 >= 0.8													
20	1796811	0.98	rs79291623	C	T					26 altered motifs		17kb 5' of *LOC100289473*	
20	1799904	1	rs113862101	C	T		BLD, GI, LNG	BLD		AP-1, BCL		20kb 5' of *LOC100289473*	

Supplementary Table S4: Description of RegulomeDB Ranks/categories based on regulatory potential.[3, 4]

Category	Description
**Likely to affect binding and linked to expression of a gene target**	
1a	eQTL + Transcription Factor (TF) binding + matched TF motif + matched DNase Footprint + DNase peak
1b	eQTL + TF binding + any motif + DNase Footprint + DNase peak
1c	eQTL + TF binding + matched TF motif + DNase peak
1d	eQTL + TF binding + any motif + DNase peak
1e	eQTL + TF binding + matched TF motif
1f	eQTL + TF binding / DNase peak
**Likely to affect binding**	
2a	TF binding + matched TF motif + matched DNase Footprint + DNase peak
2b	TF binding + any motif + DNase Footprint + DNase peak
2c	TF binding + matched TF motif + DNase peak
**Less likely to affect binding**	
3a	TF binding + any motif + DNase peak
3b	TF binding + matched TF motif
**Minimal binding evidence**	
4	TF binding + DNase peak
5	TF binding or DNase peak
6	Motif hit
7	Not available

eQTL: expression quantitative locus, Lower scores indicate increasing evidence that a variant is located in a functional regulatory region.

Supplementary Table S5: Description of FORGEdb Scores/categories based on regulatory potential.[5]

Description	Points Awarded in the presence of the feature
Expression quantitative trait locus (eQTL)	2
Activity-by-contact (ABC) contacts*	2
DNase I hotspot, marking accessible chromatin	2
Histone mark ChIP-seq broad Peak	2
Transcription factor (TF) motifs	1
Contextual analysis of TF occupancy (CATO) score	1

*Activity-By-Contact (ABC) scores enhancer-gene interactions based on enhancer activity and the contact frequency of an enhancer to its target gene.

A FORGEdb score of 10 (the highest score) is computed from the presence of all features, and a FORGEdb score of 0 (the lowest score) is computed from the absence of all features.

Supplementary Table S6: Determination of regulatory SNP (rSNP) indication, using proximal regulation, distal regulation, miRNA regulation, expression quantitative traits locus (eQTL), and regulation type from rSNPBase software.[6]

**SNP_ID**	**rSNP**	**LD-proxy of rSNP (r^2>0.8)**	**Proximal regulation**	**Distal regulation**	**miRNA regulation**	**RNA-binding protein-mediated regulation**	**eQTL**	**Regulation Type**
rs28505079	no	yes	no	no	no	no	no	
rs7233902	no	yes	no	no	no	no	no	
rs7234494	no	yes	no	no	no	no	no	
rs144650377	no	no	no	no	no	no	no	
rs1669681	no	yes	no	no	no	no	**yes**	
rs28393818	**yes**	yes	no	**yes**	no	**yes**	no	RNA-binding protein-mediated regulation; Distal transcriptional regulation
rs9944770	no	yes	no	no	no	no	no	
rs1703937	no	yes	no	no	no	no	**yes**	
rs2486958	yes	yes	**yes**	no	no	**yes**	**yes**	Proximal transcriptional regulation; RNA-binding protein-mediated regulation
rs1002485	yes	yes	**yes**	yes	no	**yes**	**yes**	Proximal transcriptional regulation; RNA-binding protein-mediated regulation; Distal transcriptional regulation
rs1556854	yes	yes	**yes**	yes	no	**yes**	no	Proximal transcriptional regulation; RNA-binding protein-mediated regulation; Distal transcriptional regulation
rs2486959	yes	yes	**yes**	yes	no	**yes**	no	Proximal transcriptional regulation; RNA-binding protein-mediated regulation; Distal transcriptional regulation
rs2244385	yes	yes	**yes**	yes	no	**yes**	no	Proximal transcriptional regulation; RNA-binding protein-mediated regulation; Distal transcriptional regulation
rs2486961	yes	yes	**yes**	no	no	**yes**	**yes**	Proximal transcriptional regulation; RNA-binding protein-mediated regulation
rs2486962	yes	yes	**yes**	no	no	**yes**	**yes**	Proximal transcriptional regulation; RNA-binding protein-mediated regulation
rs3216011	yes	no	**yes**	no	no	**yes**	no	Proximal transcriptional regulation; RNA-binding protein-mediated regulation
rs2486963	yes	yes	**yes**	no	no	**yes**	**yes**	Proximal transcriptional regulation; RNA-binding protein-mediated regulation
rs1417150	yes	yes	no	no	no	**yes**	no	RNA-binding protein-mediated regulation
rs28373933	no	yes	no	no	no	no	no	
rs28580582	no	yes	no	no	no	no	no	

SNP_ID	rSNP	LD-proxy of rSNP (r^2>0.8)	Proximal regulation	Distal regulation	miRNA regulation	RNA-binding protein-mediated regulation	eQTL	Regulation Type
rs56756677	no	yes	no	no	no	no	no	
rs28459797	no	yes	no	no	no	no	no	
rs28759291	no	yes	no	no	no	no	no	
rs17082653	no	yes	no	no	no	no	**yes**	
rs9953912	no	yes	no	no	no	no	no	
rs9964993	no	yes	no	no	no	no	no	
rs9965095	no	no	no	no	no	no	no	
rs58830422	no	yes	no	no	no	no	no	
rs9956900	no	yes	no	no	no	no	no	
rs8093705	no	yes	no	no	no	no	**yes**	
rs8092963	no	yes	no	no	no	no	no	
rs73459349	no	yes	no	no	no	no	no	
rs73459351	no	yes	no	no	no	no	no	
rs60088432	no	yes	no	no	no	no	no	
rs7234487	no	yes	no	no	no	no	no	
rs7234834	no	yes	no	no	no	no	no	
rs11996757	no	yes	no	no	no	no	no	
rs1669691	yes	yes	**yes**	no	no	no	no	Proximal transcriptional regulation
rs1669707	yes	yes	**yes**	no	no	no	no	Proximal transcriptional regulation
rs6996811	no	yes	no	no	no	no	no	
rs4735900	yes	yes	no	**yes**	no	no	**yes**	Distal transcriptional regulation
rs6993769	yes	yes	no	**yes**	no	**yes**	**yes**	RNA-binding protein-mediated regulation; Distal transcriptional regulation
rs7238797	yes	yes	no	no	no	**yes**	no	RNA-binding protein-mediated regulation
rs113862101	no	no	no	no	no	no	no	
rs77791547	yes	no	no	no	no	**yes**	no	RNA-binding protein-mediated regulation
rs60914690	yes	no	no	**yes**	no	no	no	Distal transcriptional regulation
rs73375993	yes	no	no	no	no	**yes**	no	RNA-binding protein-mediated regulation
rs80328971	yes	no	no	no	no	**yes**	no	RNA-binding protein-mediated regulation
rs13390546	yes	yes	no	no	no	**yes**	no	RNA-binding protein-mediated regulation
rs113164730	yes	no	no	no	no	**yes**	no	RNA-binding protein-mediated regulation
rs10208306	yes	yes	no	no	no	**yes**	no	RNA-binding protein-mediated regulation
rs13421314	yes	yes	no	no	no	**yes**	no	RNA-binding protein-mediated regulation
rs6735852	yes	yes	no	no	no	**yes**	no	RNA-binding protein-mediated regulation
rs113237398	yes	no	yes	no	no	no	no	Proximal transcriptional regulation
rs144396310	no	no	no	no	no	no	no	

SNP_ID	rSNP	LD-proxy of rSNP (r^2>0.8)	Proximal regulation	Distal regulation	miRNA regulation	RNA-binding protein-mediated regulation	eQTL	Regulation Type
rs72784472	no	no	no	no	no	no	no	
rs74904880	yes	no	no	no	no	**yes**	no	RNA-binding protein-mediated regulation
rs10175802	yes	yes	no	no	no	**yes**	**yes**	RNA-binding protein-mediated regulation
rs112576297	no	no	no	no	no	no	no	
rs79232623	yes	no	no	no	no	**yes**	no	RNA-binding protein-mediated regulation
rs10211451	yes	yes	no	no	no	**yes**	no	RNA-binding protein-mediated regulation
rs77158497	yes	no	no	no	no	**yes**	no	RNA-binding protein-mediated regulation
rs112614653	no	no	no	no	no	no	no	
rs10474933	yes	no	no	no	no	**yes**	no	RNA-binding protein-mediated regulation
rs1979248	no	no	no	no	no	no	no	
rs116039677	no	no	no	no	no	no	no	
rs17063846	no	yes	no	no	no	no	no	
rs28530774	yes	yes	no	no	no	**yes**	no	RNA-binding protein-mediated regulation
rs6999003	no	yes	no	no	no	no	no	
rs71517216	no	yes	no	no	no	no	no	
rs6472431	no	no	no	no	no	no	no	
rs66891172	no	no	no	no	no	no	no	
rs73459320	no	yes	no	no	no	no	no	
rs111352946	no	no	no	no	no	no	no	
rs8092458	no	yes	no	no	no	no	no	
rs66953037	yes	no	no	no	no	**yes**	no	RNA-binding protein-mediated regulation
rs1922198	no	no	no	no	no	no	no	
rs79291623	no	no	no	no	no	no	no	
rs4737926	no	no	no	no	no	no	no	
rs821932	yes	yes	yes	no	no	**yes**	no	Proximal transcriptional regulation; RNA-binding protein-mediated regulation
rs1358874	yes	yes	no	no	no	**yes**	no	RNA-binding protein-mediated regulation
rs7232674	no	yes	no	no	no	no	no	
rs8092443	no	yes	no	no	no	no	no	
rs9954746	no	yes	no	no	no	no	no	
rs35794277	no	yes	no	no	no	no	no	
rs7240698	no	yes	no	no	no	no	no	
rs112880022	yes	no	no	no	no	**yes**	no	RNA-binding protein-mediated regulation
rs79445350	yes	no	no	no	no	**yes**	no	RNA-binding protein-mediated regulation

SNP_ID	rSNP	LD-proxy of rSNP (r^2>0.8)	Proximal regulation	Distal regulation	miRNA regulation	RNA-binding protein-mediated regulation	eQTL	Regulation Type
rs13382715	yes	yes	no	no	no	**yes**	no	RNA-binding protein-mediated regulation
rs17008540	yes	yes	no	no	no	**yes**	no	RNA-binding protein-mediated regulation
rs4702814	yes	no	no	no	no	**yes**	no	RNA-binding protein-mediated regulation
rs7829447	no	yes	no	no	no	no	no	
rs6998830	no	yes	no	no	no	no	no	
rs34207383	no	yes	no	no	no	no	no	
rs147607144	no	no	no	no	no	no	no	
rs60283435	no	yes	no	no	no	no	no	
rs7001684	no	yes	no	no	no	no	**yes**	
rs4737928	no	no	no	no	no	no	no	

rSNP: regulatory Single Nucleotide Polymorphism

Supplementary Table S7: RegulomeDB, FORGEdb, and rSNPBase Scores for all 98 SNPs.[3-6]

dbSNP IDs	RegulomeDB Rank	FORGEdb Score	rSNP
rs28505079	1b	7	no
rs7233902	1b	6	no
rs7234494	1b	6	no
rs144650377	1b	2	no
rs1669681	1b	7	no
rs28393818	1b	8	yes
rs9944770	1d	8	no
rs1703937	1d	7	no
rs2486958	1f	6	yes
rs1002485	1f	6	yes
rs1556854	1f	6	yes
rs2486959	1f	6	yes
rs2244385	1f	8	yes
rs2486961	1f	6	yes
rs2486962	1f	6	yes
rs3216011	1f	2	yes
rs2486963	1f	8	yes
rs1417150	1f	6	yes
rs28373933	1f	8	no
rs28580582	1f	7	no
rs56756677	1f	8	no
rs28459797	1f	7	no
rs28759291	1f	6	no
rs17082653	1f	7	no
rs9953912	1f	6	no
rs9964993	1f	6	no
rs9965095	1f	5	no
rs58830422	1f	7	no
rs9956900	1f	5	no
rs8093705	1f	6	no
rs8092963	1f	6	no
rs73459349	1f	7	no
rs73459351	1f	6	no
rs60088432	1f	5	no
rs7234487	1f	4	no
rs7234834	1f	6	no
rs11996757	1f	7	no
rs1669691	1f	10	yes
rs1669707	1f	8	yes
rs6996811	1f	8	no
rs4735900	1f	7	yes
rs6993769	1f	8	yes
rs7238797	2a	8	yes
rs113862101	2b	5	no
rs77791547	3a	4	yes

dbSNP IDs	RegulomeDB Rank	FORGEdb Score	rSNP
rs60914690	3a	NA	yes
rs73375993	4	4	yes
rs80328971	4	4	yes
rs13390546	4	4	yes
rs113164730	4	5	yes
rs10208306	4	4	yes
rs13421314	4	5	yes
rs6735852	4	5	yes
rs113237398	4	5	yes
rs144396310	5	NA	no
rs72784472	5	6	no
rs74904880	5	6	yes
rs10175802	5	4	yes
rs112576297	5	NA	no
rs79232623	5	2	yes
rs10211451	5	6	yes
rs77158497	5	4	yes
rs112614653	5	NA	no
rs10474933	5	4	yes
rs1979248	5	4	no
rs116039677	5	4	no
rs17063846	5	2	no
rs28530774	5	8	yes
rs6999003	5	4	no
rs71517216	5	6	no
rs6472431	5	7	no
rs66891172	5	6	no
rs73459320	6	4	no
rs111352946	6	4	no
rs8092458	6	4	no
rs66953037	6	NA	yes
rs1922198	6	2	no
rs79291623	6	3	no
rs4737926	6	4	no
rs821932	7	3	yes
rs1358874	7	4	yes
rs7232674	7	8	no
rs8092443	7	5	no
rs9954746	7	4	no
rs35794277	7	5	no
rs7240698	7	5	no
rs112880022	7	5	yes
rs79445350	7	4	yes
rs13382715	7	2	yes
rs17008540	7	4	yes
rs4702814	7	4	yes
rs7829447	7	6	no

dbSNP IDs	RegulomeDB Rank	FORGEdb Score	rSNP
rs6998830	7	2	no
rs34207383	7	4	no
rs147607144	7	NA	no
rs60283435	7	4	no
rs7001684	7	4	no
rs4737928	7	3	no

Supplementary Table S8. Selected SNPs overlapping with regulatory chromatin states from DNase I hypersensitivity sites (DHS) and histone marks from the ChIP-Seq database. (**Black** = missing data). [2]

**rs2486963 (*CHIT1*)**										
**Epigenome ID (EID)**	**Group**	**Mnemonic**	**Description**	**Chromatin states (Core 15-state model)**	**Chromatin states (25-state model using 12 imputed marks)**	**H3K4me1**	**H3K4me3**	**H3K27ac**	**H3K9ac**	**DNase**
E005	ES-deriv	ESDR.H1.BMP4.TROP	H1 BMP4 Derived Trophoblast Cultured Cells						H3K9ac_Pro	
E007	ES-deriv	ESDR.H1.NEUR.PROG	H1 Derived Neuronal Progenitor Cultured Cells			H3K4me1_Enh			H3K9ac_Pro	
E018	iPSC	IPSC.15b	iPS-15b Cells						H3K9ac_Pro	
E027	Epithelial	BRST.MYO	Breast Myoepithelial Primary Cells						H3K9ac_Pro	
E030	HSC & B-cell	BLD.CD15.PC	Primary neutrophils from peripheral blood	7_Enh	22_PromP	H3K4me1_Enh				
E036	HSC & B-cell	BLD.CD34.CC	Primary hematopoietic stem cells short term culture	7_Enh						
E056	Epithelial	SKIN.PEN.FRSK.FIB.02	Foreskin Fibroblast Primary Cells skin02			H3K4me1_Enh				
E059	Epithelial	SKIN.PEN.FRSK.MEL.01	Foreskin Melanocyte Primary Cells skin01					H3K27ac_Enh		
E089	Muscle	MUS.TRNK.FET	Fetal Muscle Trunk			H3K4me1_Enh				
E090	Muscle	MUS.LEG.FET	Fetal Muscle Leg			H3K4me1_Enh				
E093	Thymus	THYM.FET	Fetal Thymus			H3K4me1_Enh		H3K27ac_Enh		
E094	Digestive	GI.STMC.GAST	Gastric	1_TssA			H3K4me3_Pro			
E108	Muscle	MUS.SKLT.F	Skeletal Muscle Female						H3K9ac_Pro	

E120	ENCODE 2012	MUS.HSMM	HSMM Skeletal Muscle Myoblasts Cells	7_Enh		H3K4me1_Enh				
E121	ENCODE 2012	MUS.HSMMT	HSMM cell derived Skeletal Muscle Myotubes Cells	7_Enh	23_PromBiv	H3K4me1_Enh		H3K27ac_Enh	H3K9ac_Pro	
**rs2244385 (*CHIT1*)**										
**Epigenome ID (EID)**	**Group**	**Mnemonic**	**Description**	**Chromatin states (Core 15-state model)**	**Chromatin states (25-state model using 12 imputed marks)**	**H3K4me1**	**H3K4me3**	**H3K27ac**	**H3K9ac**	**DNase**
E022	iPSC	IPSC.DF.19.11	iPS DF 19.11 Cells			H3K4me1_Enh		H3K27ac_Enh		
E027	Epithelial	BRST.MYO	Breast Myoepithelial Primary Cells						H3K9ac_Pro	
E030	HSC & B-cell	BLD.CD15.PC	Primary neutrophils from peripheral blood	7_Enh		H3K4me1_Enh				
E055	Epithelial	SKIN.PEN.FRSK.FIB.01	Foreskin Fibroblast Primary Cells skin01					H3K27ac_Enh		
E056	Epithelial	SKIN.PEN.FRSK.FIB.02	Foreskin Fibroblast Primary Cells skin02			H3K4me1_Enh				
E074	Brain	BRN.SUB.NIG	Brain Substantia Nigra				H3K4me3_Pro			
E093	Thymus	THYM.FET	Fetal Thymus			H3K4me1_Enh				
E103	Sm. Muscle	GI.RECT.SM.MUS	Rectal Smooth Muscle						H3K9ac_Pro	
E113	Other	SPLN	Spleen			H3K4me1_Enh	H3K4me3_Pro			
E124	ENCODE 2012	BLD.CD14.MONO	Monocytes-CD14+ RO01746 Primary Cells						H3K9ac_Pro	
**rs1669691 (1.2kb 5' of *TDRP*)**										
**Epigenome ID (EID)**	**Group**	**Mnemonic**	**Description**	**Chromatin states**	**Chromatin states**	**H3K4me1**	**H3K4me3**	**H3K27ac**	**H3K9ac**	**DNase**

				(Core 15-state model)	(25-state model using 12 imputed marks)					
E001	ESC	ESC.I3	ES-I3 Cells	10_TssBiv	23_PromBiv		H3K4me3_Pro		H3K9ac_Pro	
E002	ESC	ESC.WA7	ES-WA7 Cells	1_TssA	23_PromBiv		H3K4me3_Pro			
E003	ESC	ESC.H1	H1 Cells	11_BivFlnk	23_PromBiv	H3K4me1_Enh	H3K4me3_Pro		H3K9ac_Pro	
E004	ES-deriv	ESDR.H1.BMP4.MESO	H1 BMP4 Derived Mesendoderm Cultured Cells	1_TssA	23_PromBiv	H3K4me1_Enh	H3K4me3_Pro	H3K27ac_Enh	H3K9ac_Pro	DNase
E005	ES-deriv	ESDR.H1.BMP4.TROP	H1 BMP4 Derived Trophoblast Cultured Cells	2_TssAFlnk	16_EnhW1	H3K4me1_Enh	H3K4me3_Pro	H3K27ac_Enh		
E006	ES-deriv	ESDR.H1.MSC	H1 Derived Mesenchymal Stem Cells	7_Enh	16_EnhW1	H3K4me1_Enh			H3K9ac_Pro	
E007	ES-deriv	ESDR.H1.NEUR.PROG	H1 Derived Neuronal Progenitor Cultured Cells	7_Enh	23_PromBiv	H3K4me1_Enh	H3K4me3_Pro		H3K9ac_Pro	DNase
E008	ESC	ESC.H9	H9 Cells	1_TssA	23_PromBiv		H3K4me3_Pro		H3K9ac_Pro	
E009	ES-deriv	ESDR.H9.NEUR.PROG	H9 Derived Neuronal Progenitor Cultured Cells	2_TssAFlnk	23_PromBiv	H3K4me1_Enh	H3K4me3_Pro			
E010	ES-deriv	ESDR.H9.NEUR	H9 Derived Neuron Cultured Cells	2_TssAFlnk	23_PromBiv	H3K4me1_Enh	H3K4me3_Pro			
E011	ES-deriv	ESDR.CD184.ENDO	hESC Derived CD184+ Endoderm Cultured Cells	1_TssA	23_PromBiv	H3K4me1_Enh	H3K4me3_Pro		H3K9ac_Pro	
E012	ES-deriv	ESDR.CD56.ECTO	hESC Derived CD56+ Ectoderm Cultured Cells	11_BivFlnk	23_PromBiv	H3K4me1_Enh	H3K4me3_Pro	H3K27ac_Enh		
E013	ES-deriv	ESDR.CD56.MESO	hESC Derived CD56+ Mesoderm Cultured Cells	1_TssA	23_PromBiv	H3K4me1_Enh	H3K4me3_Pro	H3K27ac_Enh		
E014	ESC	ESC.HUES48	HUES48 Cells	11_BivFlnk	23_PromBiv	H3K4me1_Enh	H3K4me3_Pro	H3K27ac_Enh	H3K9ac_Pro	
E015	ESC	ESC.HUES6	HUES6 Cells	2_TssAFlnk	23_PromBiv	H3K4me1_Enh	H3K4me3_Pro	H3K27ac_Enh	H3K9ac_Pro	

E016	ESC	ESC.HUES64	HUES64 Cells	10_TssBiv	23_PromBiv	H3K4me1_Enh	H3K4me3_Pro	H3K27ac_Enh	H3K9ac_Pro	
E017	IMR90	LNG.IMR90	IMR90 fetal lung fibroblasts Cell Line		16_EnhW1	H3K4me1_Enh	H3K4me3_Pro		H3K9ac_Pro	
E018	iPSC	IPSC.15b	iPS-15b Cells	1_TssA	23_PromBiv		H3K4me3_Pro		H3K9ac_Pro	
E019	iPSC	IPSC.18	iPS-18 Cells	11_BivFlnk	23_PromBiv	H3K4me1_Enh	H3K4me3_Pro		H3K9ac_Pro	
E020	iPSC	IPSC.20B	iPS-20b Cells	10_TssBiv	23_PromBiv	H3K4me1_Enh	H3K4me3_Pro		H3K9ac_Pro	
E021	iPSC	IPSC.DF.6.9	iPS DF 6.9 Cells	1_TssA	23_PromBiv		H3K4me3_Pro			
E022	iPSC	IPSC.DF.19.11	iPS DF 19.11 Cells	2_TssAFlnk	23_PromBiv	H3K4me1_Enh	H3K4me3_Pro			
E024	ESC	ESC.4STAR	ES-UCSF4 Cells	2_TssAFlnk	2_PromU	H3K4me1_Enh	H3K4me3_Pro			
E026	Mesench	STRM.MRW.MSC	Bone Marrow Derived Cultured Mesenchymal Stem Cells				H3K4me3_Pro			
E027	Epithelial	BRST.MYO	Breast Myoepithelial Primary Cells	1_TssA	22_PromP	H3K4me1_Enh	H3K4me3_Pro		H3K9ac_Pro	
E028	Epithelial	BRST.HMEC.35	Breast variant Human Mammary Epithelial Cells (vHMEC)		16_EnhW1		H3K4me3_Pro			
E039	Blood & T-cell	BLD.CD4.CD25M.CD45RA.NPC	Primary T helper naive cells from peripheral blood			H3K4me1_Enh				
E045	Blood & T-cell	BLD.CD4.CD25I.CD127.TMEMPC	Primary T cells effector/memory enriched from peripheral blood			H3K4me1_Enh				
E049	Mesench	STRM.CHON.MRW.DR.MSC	Mesenchymal Stem Cell Derived Chondrocyte Cultured Cells		22_PromP	H3K4me1_Enh			H3K9ac_Pro	
E050	HSC & B-cell	BLD.MOB.CD34.PC.F	Primary hematopoietic stem cells G-CSF-mobilized Female			H3K4me1_Enh				
E052	Myosat	MUS.SAT	Muscle Satellite Cultured Cells		22_PromP		H3K4me3_Pro		H3K9ac_Pro	
E053	Neurosph	BRN.CRTX.DR.NRSPHR	Cortex derived primary cultured neurospheres	2_TssAFlnk	2_PromU	H3K4me1_Enh	H3K4me3_Pro			

E054	Neurosph	BRN.GANGEM.DR.NRSPHR	Ganglion Eminence derived primary cultured neurospheres	2_TssAFlnk	2_PromU	H3K4me1_Enh	H3K4me3_Pro			
E055	Epithelial	SKIN.PEN.FRSK.FIB.01	Foreskin Fibroblast Primary Cells skin01	2_TssAFlnk	2_PromU	H3K4me1_Enh	H3K4me3_Pro			
E056	Epithelial	SKIN.PEN.FRSK.FIB.02	Foreskin Fibroblast Primary Cells skin02	2_TssAFlnk	16_EnhW1	H3K4me1_Enh	H3K4me3_Pro	H3K27ac_Enh		
E057	Epithelial	SKIN.PEN.FRSK.KER.02	Foreskin Keratinocyte Primary Cells skin02	1_TssA	16_EnhW1		H3K4me3_Pro			
E058	Epithelial	SKIN.PEN.FRSK.KER.03	Foreskin Keratinocyte Primary Cells skin03	2_TssAFlnk	2_PromU	H3K4me1_Enh	H3K4me3_Pro	H3K27ac_Enh		
E059	Epithelial	SKIN.PEN.FRSK.MEL.01	Foreskin Melanocyte Primary Cells skin01		16_EnhW1		H3K4me3_Pro			
E061	Epithelial	SKIN.PEN.FRSK.MEL.03	Foreskin Melanocyte Primary Cells skin03	2_TssAFlnk	2_PromU	H3K4me1_Enh	H3K4me3_Pro	H3K27ac_Enh		
E062	Blood & T-cell	BLD.PER.MONUC.PC	Primary mononuclear cells from peripheral blood				H3K4me3_Pro			
E063	Adipose	FAT.ADIP.NUC	Adipose Nuclei		22_PromP	H3K4me1_Enh		H3K27ac_Enh	H3K9ac_Pro	
E065	Heart	VAS.AOR	Aorta				H3K4me3_Pro			
E066	Other	LIV.ADLT	Liver		22_PromP	H3K4me1_Enh		H3K27ac_Enh	H3K9ac_Pro	
E067	Brain	BRN.ANG.GYR	Brain Angular Gyrus		16_EnhW1	H3K4me1_Enh	H3K4me3_Pro	H3K27ac_Enh	H3K9ac_Pro	
E068	Brain	BRN.ANT.CAUD	Brain Anterior Caudate	7_Enh	14_EnhA2	H3K4me1_Enh	H3K4me3_Pro	H3K27ac_Enh	H3K9ac_Pro	
E069	Brain	BRN.CING.GYR	Brain Cingulate Gyrus	2_TssAFlnk	2_PromU	H3K4me1_Enh	H3K4me3_Pro	H3K27ac_Enh	H3K9ac_Pro	
E070	Brain	BRN.GRM.MTRX	Brain Germinal Matrix	2_TssAFlnk	2_PromU		H3K4me3_Pro			
E071	Brain	BRN.HIPP.MID	Brain Hippocampus Middle	2_TssAFlnk	2_PromU	H3K4me1_Enh	H3K4me3_Pro	H3K27ac_Enh		
E072	Brain	BRN.INF.TMP	Brain Inferior Temporal Lobe	1_TssA	16_EnhW1	H3K4me1_Enh	H3K4me3_Pro	H3K27ac_Enh	H3K9ac_Pro	
E073	Brain	BRN.DL.PRFRNTL.CRTX	Brain_Dorsolateral_Prefrontal_Cortex	7_Enh	16_EnhW1			H3K27ac_Enh	H3K9ac_Pro	

E074	Brain	BRN.SUB.NIG	Brain Substantia Nigra		16_EnhW1	H3K4me1_Enh	H3K4me3_Pro	H3K27ac_Enh	H3K9ac_Pro	
E075	Digestive	GI.CLN.MUC	Colonic Mucosa		23_PromBiv	H3K4me1_Enh	H3K4me3_Pro	H3K27ac_Enh	H3K9ac_Pro	
E076	Sm. Muscle	GI.CLN.SM.MUS	Colon Smooth Muscle		16_EnhW1		H3K4me3_Pro	H3K27ac_Enh	H3K9ac_Pro	
E078	Sm. Muscle	GI.DUO.SM.MUS	Duodenum Smooth Muscle		23_PromBiv		H3K4me3_Pro			
E080	Other	ADRL.GLND.FET	Fetal Adrenal Gland	12_EnhBiv	23_PromBiv	H3K4me1_Enh	H3K4me3_Pro	H3K27ac_Enh		
E081	Brain	BRN.FET.M	Fetal Brain Male	7_Enh	2_PromU	H3K4me1_Enh				
E082	Brain	BRN.FET.F	Fetal Brain Female	1_TssA	2_PromU	H3K4me1_Enh	H3K4me3_Pro			
E083	Heart	HRT.FET	Fetal Heart	7_Enh	23_PromBiv	H3K4me1_Enh			H3K9ac_Pro	
E084	Digestive	GI.L.INT.FET	Fetal Intestine Large		23_PromBiv		H3K4me3_Pro			
E085	Digestive	GI.S.INT.FET	Fetal Intestine Small	10_TssBiv	23_PromBiv	H3K4me1_Enh	H3K4me3_Pro	H3K27ac_Enh		
E086	Other	KID.FET	Fetal Kidney		16_EnhW1		H3K4me3_Pro		H3K9ac_Pro	
E087	Other	PANC.ISLT	Pancreatic Islets					H3K27ac_Enh	H3K9ac_Pro	
E088	Other	LNG.FET	Fetal Lung	7_Enh	2_PromU	H3K4me1_Enh	H3K4me3_Pro		H3K9ac_Pro	DNase
E089	Muscle	MUS.TRNK.FET	Fetal Muscle Trunk	12_EnhBiv	2_PromU	H3K4me1_Enh	H3K4me3_Pro	H3K27ac_Enh		DNase
E090	Muscle	MUS.LEG.FET	Fetal Muscle Leg	2_TssAFlnk	2_PromU	H3K4me1_Enh	H3K4me3_Pro	H3K27ac_Enh		
E091	Other	PLCNT.FET	Placenta	7_Enh	23_PromBiv	H3K4me1_Enh	H3K4me3_Pro	H3K27ac_Enh		
E092	Digestive	GI.STMC.FET	Fetal Stomach	2_TssAFlnk	2_PromU	H3K4me1_Enh	H3K4me3_Pro	H3K27ac_Enh		
E093	Thymus	THYM.FET	Fetal Thymus				H3K4me3_Pro			
E094	Digestive	GI.STMC.GAST	Gastric		22_PromP	H3K4me1_Enh		H3K27ac_Enh		

E095	Heart	HRT.VENT.L	Left Ventricle				H3K4me3_Pro			
E096	Other	LNG	Lung				H3K4me3_Pro	H3K27ac_Enh		
**E097**	**Other**	**OVRY**	**Ovary**	1_TssA	16_EnhW1	H3K4me1_Enh	H3K4me3_Pro	H3K27ac_Enh		
E098	Other	PANC	Pancreas	7_Enh	22_PromP	H3K4me1_Enh	H3K4me3_Pro			
E099	Other	PLCNT.AMN	Placenta Amnion	7_Enh	16_EnhW1	H3K4me1_Enh				
E100	Muscle	MUS.PSOAS	Psoas Muscle		17_EnhW2		H3K4me3_Pro	H3K27ac_Enh		
E101	Digestive	GI.RECT.MUC.29	Rectal Mucosa Donor 29				H3K4me3_Pro	H3K27ac_Enh		
E102	Digestive	GI.RECT.MUC.31	Rectal Mucosa Donor 31				H3K4me3_Pro			
E103	Sm. Muscle	GI.RECT.SM.MUS	Rectal Smooth Muscle		16_EnhW1		H3K4me3_Pro	H3K27ac_Enh	H3K9ac_Pro	
E104	Heart	HRT.ATR.R	Right Atrium		22_PromP	H3K4me1_Enh	H3K4me3_Pro	H3K27ac_Enh		
E105	Heart	HRT.VNT.R	Right Ventricle		16_EnhW1	H3K4me1_Enh	H3K4me3_Pro	H3K27ac_Enh		
E106	Digestive	GI.CLN.SIG	Sigmoid Colon					H3K27ac_Enh		
E107	Muscle	MUS.SKLT.M	Skeletal Muscle Male				H3K4me3_Pro			
E108	Muscle	MUS.SKLT.F	Skeletal Muscle Female		17_EnhW2			H3K27ac_Enh		
E109	Digestive	GI.S.INT	Small Intestine			H3K4me1_Enh	H3K4me3_Pro	H3K27ac_Enh		
E110	Digestive	GI.STMC.MUC	Stomach Mucosa							
E111	Sm. Muscle	GI.STMC.MUS	Stomach Smooth Muscle	1_TssA	16_EnhW1	H3K4me1_Enh	H3K4me3_Pro	H3K27ac_Enh	H3K9ac_Pro	
E112	Thymus	THYM	Thymus				H3K4me3_Pro			
E113	Other	SPLN	Spleen				H3K4me3_Pro	H3K27ac_Enh		
E114	ENCODE 2012	LNG.A549.ETOH002.CNCR	A549 EtOH 0.02pct Lung Carcinoma Cell Line	2_TssAFlnk	23_PromBiv	H3K4me1_Enh	H3K4me3_Pro		H3K9ac_Pro	

E118	ENCODE 2012	LIV.HEPG2.CNCR	HepG2 Hepatocellular Carcinoma Cell Line	11_BivFlnk	23_PromBiv	H3K4me1_Enh	H3K4me3_Pro		H3K9ac_Pro	
E119	ENCODE 2012	BRST.HMEC	HMEC Mammary Epithelial Primary Cells		23_PromBiv				H3K9ac_Pro	
E120	ENCODE 2012	MUS.HSMM	HSMM Skeletal Muscle Myoblasts Cells	7_Enh	22_PromP	H3K4me1_Enh			H3K9ac_Pro	
E121	ENCODE 2012	MUS.HSMMT	HSMM cell derived Skeletal Muscle Myotubes Cells	7_Enh	22_PromP	H3K4me1_Enh	H3K4me3_Pro	H3K27ac_Enh	H3K9ac_Pro	
E125	ENCODE 2012	BRN.NHA	NH-A Astrocytes Primary Cells		23_PromBiv	H3K4me1_Enh	H3K4me3_Pro	H3K27ac_Enh	H3K9ac_Pro	
E127	ENCODE 2012	SKIN.NHEK	NHEK-Epidermal Keratinocyte Primary Cells		16_EnhW1		H3K4me3_Pro			
E129	ENCODE 2012	BONE.OSTEO	Osteoblast Primary Cells		22_PromP		H3K4me3_Pro			

rs1669707 (1.9kb 5' of *TDRP*)

Epigenome ID (EID)	Group	Mnemonic	Description	Chromatin states (Core 15-state model)	Chromatin states (25-state model using 12 imputed marks)	H3K4me1	H3K4me3	H3K27ac	H3K9ac	DNase
E001	ESC	ESC.I3	ES-I3 Cells	12_EnhBiv	23_PromBiv	H3K4me1_Enh	H3K4me3_Pro		H3K9ac_Pro	
E002	ESC	ESC.WA7	ES-WA7 Cells			H3K4me1_Enh	H3K4me3_Pro			
E003	ESC	ESC.H1	H1 Cells			H3K4me1_Enh			H3K9ac_Pro	
E004	ES-deriv	ESDR.H1.BMP4.MESO	H1 BMP4 Derived Mesendoderm Cultured Cells			H3K4me1_Enh		H3K27ac_Enh	H3K9ac_Pro	
**E005**	**ES-deriv**	**ESDR.H1.BMP4.TROP**	**H1 BMP4 Derived Trophoblast Cultured Cells**			H3K4me1_Enh		H3K27ac_Enh		
E007	ES-deriv	ESDR.H1.NEUR.PROG	H1 Derived Neuronal Progenitor Cultured Cells			H3K4me1_Enh				DNase

E008	ESC	ESC.H9	H9 Cells				H3K4me3_Pro		H3K9ac_Pro	DNase
E009	ES-deriv	ESDR.H9.NEUR.PROG	H9 Derived Neuronal Progenitor Cultured Cells			H3K4me1_Enh	H3K4me3_Pro			
E010	ES-deriv	ESDR.H9.NEUR	H9 Derived Neuron Cultured Cells			H3K4me1_Enh	H3K4me3_Pro			
E011	ES-deriv	ESDR.CD184.ENDO	hESC Derived CD184+ Endoderm Cultured Cells	12_EnhBiv	23_PromBiv	H3K4me1_Enh	H3K4me3_Pro		H3K9ac_Pro	
E012	ES-deriv	ESDR.CD56.ECTO	hESC Derived CD56+ Ectoderm Cultured Cells			H3K4me1_Enh	H3K4me3_Pro	H3K27ac_Enh		
E013	ES-deriv	ESDR.CD56.MESO	hESC Derived CD56+ Mesoderm Cultured Cells	7_Enh	17_EnhW2	H3K4me1_Enh		H3K27ac_Enh		
E014	ESC	ESC.HUES48	HUES48 Cells	11_BivFlnk	23_PromBiv	H3K4me1_Enh	H3K4me3_Pro	H3K27ac_Enh	H3K9ac_Pro	
E015	ESC	ESC.HUES6	HUES6 Cells	7_Enh		H3K4me1_Enh	H3K4me3_Pro			
E016	ESC	ESC.HUES64	HUES64 Cells	12_EnhBiv		H3K4me1_Enh			H3K9ac_Pro	
E018	iPSC	IPSC.15b	iPS-15b Cells	12_EnhBiv	23_PromBiv	H3K4me1_Enh	H3K4me3_Pro			
E019	iPSC	IPSC.18	iPS-18 Cells	12_EnhBiv	23_PromBiv	H3K4me1_Enh	H3K4me3_Pro		H3K9ac_Pro	
E020	iPSC	IPSC.20B	iPS-20b Cells	12_EnhBiv	23_PromBiv	H3K4me1_Enh	H3K4me3_Pro			
E021	iPSC	IPSC.DF.6.9	iPS DF 6.9 Cells		17_EnhW2	H3K4me1_Enh				DNase
E022	iPSC	IPSC.DF.19.11	iPS DF 19.11 Cells			H3K4me1_Enh				
E024	ESC	ESC.4STAR	ES-UCSF4 Cells	7_Enh		H3K4me1_Enh	H3K4me3_Pro			
E027	Epithelial	BRST.MYO	Breast Myoepithelial Primary Cells			H3K4me1_Enh	H3K4me3_Pro			
E028	Epithelial	BRST.HMEC.35	Breast variant Human Mammary Epithelial Cells (vHMEC)				H3K4me3_Pro			
E052	Myosat	MUS.SAT	Muscle Satellite Cultured Cells				H3K4me3_Pro			

E055	Epithelial	SKIN.PEN.FRSK.FIB.01	Foreskin Fibroblast Primary Cells skin01			H3K4me1_Enh				
E056	Epithelial	SKIN.PEN.FRSK.FIB.02	Foreskin Fibroblast Primary Cells skin02			H3K4me1_Enh				
E058	Epithelial	SKIN.PEN.FRSK.KER.03	Foreskin Keratinocyte Primary Cells skin03					H3K27ac_Enh		
E059	Epithelial	SKIN.PEN.FRSK.MEL.01	Foreskin Melanocyte Primary Cells skin01							DNase
E061	Epithelial	SKIN.PEN.FRSK.MEL.03	Foreskin Melanocyte Primary Cells skin03		22_PromP	H3K4me1_Enh	H3K4me3_Pro	H3K27ac_Enh		
E063	Adipose	FAT.ADIP.NUC	Adipose Nuclei					H3K27ac_Enh	H3K9ac_Pro	
E066	Other	LIV.ADLT	Liver			H3K4me1_Enh			H3K9ac_Pro	
E067	Brain	BRN.ANG.GYR	Brain Angular Gyrus				H3K4me3_Pro	H3K27ac_Enh		
E068	Brain	BRN.ANT.CAUD	Brain Anterior Caudate						H3K9ac_Pro	
E069	Brain	BRN.CING.GYR	Brain Cingulate Gyrus				H3K4me3_Pro	H3K27ac_Enh		
E071	Brain	BRN.HIPP.MID	Brain Hippocampus Middle				H3K4me3_Pro	H3K27ac_Enh		
E072	Brain	BRN.INF.TMP	Brain Inferior Temporal Lobe				H3K4me3_Pro	H3K27ac_Enh	H3K9ac_Pro	
E073	Brain	BRN.DL.PRFRNTL.CRTX	Brain_Dorsolateral_Prefrontal_Cortex					H3K27ac_Enh		
E074	Brain	BRN.SUB.NIG	Brain Substantia Nigra			H3K4me1_Enh		H3K27ac_Enh	H3K9ac_Pro	
E075	Digestive	GI.CLN.MUC	Colonic Mucosa				H3K4me3_Pro			
E076	Sm. Muscle	GI.CLN.SM.MUS	Colon Smooth Muscle						H3K9ac_Pro	
E080	Other	ADRL.GLND.FET	Fetal Adrenal Gland			H3K4me1_Enh				
E081	Brain	BRN.FET.M	Fetal Brain Male			H3K4me1_Enh				
E082	Brain	BRN.FET.F	Fetal Brain Female			H3K4me1_Enh				
E083	Heart	HRT.FET	Fetal Heart		17_EnhW2	H3K4me1_Enh			H3K9ac_Pro	DNase

E085	Digestive	GI.S.INT.FET	Fetal Intestine Small			H3K4me1_Enh				
E086	Other	KID.FET	Fetal Kidney				H3K4me3_Pro		H3K9ac_Pro	
E088	Other	LNG.FET	Fetal Lung	7_Enh		H3K4me1_Enh	H3K4me3_Pro		H3K9ac_Pro	
E089	Muscle	MUS.TRNK.FET	Fetal Muscle Trunk			H3K4me1_Enh		H3K27ac_Enh		
E090	Muscle	MUS.LEG.FET	Fetal Muscle Leg			H3K4me1_Enh	H3K4me3_Pro			
E091	Other	PLCNT.FET	Placenta					H3K27ac_Enh		
E092	Digestive	GI.STMC.FET	Fetal Stomach			H3K4me1_Enh				
E093	Thymus	THYM.FET	Fetal Thymus				H3K4me3_Pro			
E097	Other	OVRY	Ovary			H3K4me1_Enh		H3K27ac_Enh		
E101	Digestive	GI.RECT.MUC.29	Rectal Mucosa Donor 29					H3K27ac_Enh		
E103	Sm. Muscle	GI.RECT.SM.MUS	Rectal Smooth Muscle				H3K4me3_Pro			
E106	Digestive	GI.CLN.SIG	Sigmoid Colon					H3K27ac_Enh		
E109	Digestive	GI.S.INT	Small Intestine			H3K4me1_Enh				
E111	Sm. Muscle	GI.STMC.MUS	Stomach Smooth Muscle			H3K4me1_Enh		H3K27ac_Enh		
E113	Other	SPLN	Spleen					H3K27ac_Enh		
E114	ENCODE 2012	LNG.A549.ETOH002.CNCR	A549 EtOH 0.02pct Lung Carcinoma Cell Line			H3K4me1_Enh				
E118	ENCODE 2012	LIV.HEPG2.CNCR	HepG2 Hepatocellular Carcinoma Cell Line			H3K4me1_Enh				
E120	ENCODE 2012	MUS.HSMM	HSMM Skeletal Muscle Myoblasts Cells							DNase
E121	ENCODE 2012	MUS.HSMMT	HSMM cell derived Skeletal Muscle Myotubes Cells			H3K4me1_Enh	H3K4me3_Pro	H3K27ac_Enh	H3K9ac_Pro	DNase

E125	ENCODE 2012	BRN.NHA	NH-A Astrocytes Primary Cells				H3K4me3_Pro	H3K27ac_Enh		

rs6993769 (6.2kb 3' of *ERICH1*)

Epigenome ID (EID)	Group	Mnemonic	Description	Chromatin states (Core 15-state model)	Chromatin states (25-state model using 12 imputed marks)	H3K4me1	H3K4me3	H3K27ac	H3K9ac	DNase
E011	ES-deriv	ESDR.CD184.ENDO	hESC Derived CD184+ Endoderm Cultured Cells				H3K4me3_Pro			
E019	iPSC	IPSC.18	iPS-18 Cells				H3K4me3_Pro			
E029	HSC & B-cell	BLD.CD14.PC	Primary monocytes from peripheral blood					H3K27ac_Enh		
E056	Epithelial	SKIN.PEN.FRSK.FIB.02	Foreskin Fibroblast Primary Cells skin02					H3K27ac_Enh		
E059	Epithelial	SKIN.PEN.FRSK.MEL.01	Foreskin Melanocyte Primary Cells skin01					H3K27ac_Enh		
E105	Heart	HRT.VNT.R	Right Ventricle					H3K27ac_Enh		

rs28393818 (5.8kb 3' of *ERICH1*)

Epigenome ID (EID)	Group	Mnemonic	Description	Chromatin states (Core 15-state model)	Chromatin states (25-state model using 12 imputed marks)	H3K4me1	H3K4me3	H3K27ac	H3K9ac	DNase
E019	iPSC	IPSC.18	iPS-18 Cells				H3K4me3_Pro			
E029	HSC & B-cell	BLD.CD14.PC	Primary monocytes from peripheral blood					H3K27ac_Enh		
E056	Epithelial	SKIN.PEN.FRSK.FIB.02	Foreskin Fibroblast Primary Cells skin02					H3K27ac_Enh		
E059	Epithelial	SKIN.PEN.FRSK.MEL.01	Foreskin Melanocyte Primary Cells skin01					H3K27ac_Enh		

rs7238797 (*DLGAP1*)

Epigenome ID (EID)	Group	Mnemonic	Description	Chromatin states (Core 15-state model)	Chromatin states (25-state model using 12 imputed marks)	H3K4me1	H3K4me3	H3K27ac	H3K9ac	DNase
E001	ESC	ESC.I3	ES-I3 Cells	2_TssAFlnk	13_EnhA1	H3K4me1_Enh	H3K4me3_Pro		H3K9ac_Pro	
E002	ESC	ESC.WA7	ES-WA7 Cells	7_Enh	14_EnhA2	H3K4me1_Enh				
E003	ESC	ESC.H1	H1 Cells	2_TssAFlnk	13_EnhA1	H3K4me1_Enh	H3K4me3_Pro	H3K27ac_Enh		DNase
E004	ES-deriv	ESDR.H1.BMP4.MESO	H1 BMP4 Derived Mesendoderm Cultured Cells	2_TssAFlnk	13_EnhA1	H3K4me1_Enh		H3K27ac_Enh	H3K9ac_Pro	DNase
E005	ES-deriv	ESDR.H1.BMP4.TROP	H1 BMP4 Derived Trophoblast Cultured Cells		14_EnhA2	H3K4me1_Enh				DNase
E006	ES-deriv	ESDR.H1.MSC	H1 Derived Mesenchymal Stem Cells		19_DNase					
E007	ES-deriv	ESDR.H1.NEUR.PROG	H1 Derived Neuronal Progenitor Cultured Cells		14_EnhA2	H3K4me1_Enh				DNase
E008	ESC	ESC.H9	H9 Cells	2_TssAFlnk	13_EnhA1	H3K4me1_Enh	H3K4me3_Pro	H3K27ac_Enh	H3K9ac_Pro	DNase
E009	ES-deriv	ESDR.H9.NEUR.PROG	H9 Derived Neuronal Progenitor Cultured Cells	7_Enh	13_EnhA1	H3K4me1_Enh				
E010	ES-deriv	ESDR.H9.NEUR	H9 Derived Neuron Cultured Cells	7_Enh	13_EnhA1	H3K4me1_Enh				
E011	ES-deriv	ESDR.CD184.ENDO	hESC Derived CD184+ Endoderm Cultured Cells	1_TssA	2_PromU	H3K4me1_Enh	H3K4me3_Pro	H3K27ac_Enh	H3K9ac_Pro	
E012	ES-deriv	ESDR.CD56.ECTO	hESC Derived CD56+ Ectoderm Cultured Cells	7_Enh	2_PromU	H3K4me1_Enh		H3K27ac_Enh		
E013	ES-deriv	ESDR.CD56.MESO	hESC Derived CD56+ Mesoderm Cultured Cells	7_Enh	13_EnhA1	H3K4me1_Enh		H3K27ac_Enh		

E014	ESC	ESC.HUES48	HUES48 Cells	2_TssAFlnk	2_PromU	H3K4me1_Enh	H3K4me3_Pro	H3K27ac_Enh		
E015	ESC	ESC.HUES6	HUES6 Cells	2_TssAFlnk	13_EnhA1	H3K4me1_Enh	H3K4me3_Pro	H3K27ac_Enh	H3K9ac_Pro	
E016	ESC	ESC.HUES64	HUES64 Cells	2_TssAFlnk	2_PromU	H3K4me1_Enh	H3K4me3_Pro	H3K27ac_Enh	H3K9ac_Pro	
E017	IMR90	LNG.IMR90	IMR90 fetal lung fibroblasts Cell Line		19_DNase					
E018	iPSC	IPSC.15b	iPS-15b Cells	2_TssAFlnk	13_EnhA1	H3K4me1_Enh	H3K4me3_Pro		H3K9ac_Pro	
E019	iPSC	IPSC.18	iPS-18 Cells	2_TssAFlnk	2_PromU	H3K4me1_Enh	H3K4me3_Pro	H3K27ac_Enh	H3K9ac_Pro	
E020	iPSC	IPSC.20B	iPS-20b Cells	2_TssAFlnk	13_EnhA1	H3K4me1_Enh	H3K4me3_Pro	H3K27ac_Enh	H3K9ac_Pro	
E021	iPSC	IPSC.DF.6.9	iPS DF 6.9 Cells	7_Enh	14_EnhA2	H3K4me1_Enh		H3K27ac_Enh		DNase
E022	iPSC	IPSC.DF.19.11	iPS DF 19.11 Cells		14_EnhA2	H3K4me1_Enh		H3K27ac_Enh		DNase
E024	ESC	ESC.4STAR	ES-UCSF4 Cells	7_Enh	14_EnhA2	H3K4me1_Enh				
E026	Mesench	STRM.MRW.MSC	Bone Marrow Derived Cultured Mesenchymal Stem Cells		19_DNase					
E049	Mesench	STRM.CHON.MRW.DR.MSC	Mesenchymal Stem Cell Derived Chondrocyte Cultured Cells		19_DNase	H3K4me1_Enh				
E052	Myosat	MUS.SAT	Muscle Satellite Cultured Cells		19_DNase					
E053	Neurosph	BRN.CRTX.DR.NRSPHR	Cortex derived primary cultured neurospheres	7_Enh	16_EnhW1	H3K4me1_Enh				
E054	Neurosph	BRN.GANGEM.DR.NRSPHR	Ganglion Eminence derived primary cultured neurospheres	7_Enh	16_EnhW1	H3K4me1_Enh				
E055	Epithelial	SKIN.PEN.FRSK.FIB.01	Foreskin Fibroblast Primary Cells skin01		19_DNase					DNase
E056	Epithelial	SKIN.PEN.FRSK.FIB.02	Foreskin Fibroblast Primary Cells skin02		19_DNase					DNase
E059	Epithelial	SKIN.PEN.FRSK.MEL.01	Foreskin Melanocyte Primary Cells skin01		19_DNase					DNase

E061	Epithelial	SKIN.PEN.FRSK.MEL.03	Foreskin Melanocyte Primary Cells skin03		19_DNase	H3K4me1_Enh				
E066	Other	LIV.ADLT	Liver		19_DNase					
E067	Brain	BRN.ANG.GYR	Brain Angular Gyrus			H3K4me1_Enh				
E068	Brain	BRN.ANT.CAUD	Brain Anterior Caudate		22_PromP		H3K4me3_Pro			
E069	Brain	BRN.CING.GYR	Brain Cingulate Gyrus		19_DNase					
E071	Brain	BRN.HIPP.MID	Brain Hippocampus Middle			H3K4me1_Enh				
E072	Brain	BRN.INF.TMP	Brain Inferior Temporal Lobe		22_PromP			H3K27ac_Enh	H3K9ac_Pro	
E074	Brain	BRN.SUB.NIG	Brain Substantia Nigra		19_DNase	H3K4me1_Enh				
E082	Brain	BRN.FET.F	Fetal Brain Female		19_DNase					
E083	Heart	HRT.FET	Fetal Heart		15_EnhAF					DNase
E084	Digestive	GI.L.INT.FET	Fetal Intestine Large		19_DNase	H3K4me1_Enh				
E085	Digestive	GI.S.INT.FET	Fetal Intestine Small		19_DNase	H3K4me1_Enh				DNase
E086	Other	KID.FET	Fetal Kidney		19_DNase					DNase
E088	Other	LNG.FET	Fetal Lung							DNase
E089	Muscle	MUS.TRNK.FET	Fetal Muscle Trunk			H3K4me1_Enh				
E090	Muscle	MUS.LEG.FET	Fetal Muscle Leg		19_DNase	H3K4me1_Enh				
E091	Other	PLCNT.FET	Placenta							DNase
E094	Digestive	GI.STMC.GAST	Gastric		19_DNase					
E098	Other	PANC	Pancreas		19_DNase					
E099	Other	PLCNT.AMN	Placenta Amnion		19_DNase					

E113	Other	SPLN	Spleen				H3K4me3_Pro			
E114	ENCODE 2012	LNG.A549.ETOH002.CNCR	A549 EtOH 0.02pct Lung Carcinoma Cell Line		22_PromP	H3K4me1_Enh				
E117	ENCODE 2012	CRVX.HELAS3.CNCR	HeLa-S3 Cervical Carcinoma Cell Line		18_EnhAc					
E118	ENCODE 2012	LIV.HEPG2.CNCR	HepG2 Hepatocellular Carcinoma Cell Line	7_Enh	19_DNase					
E120	ENCODE 2012	MUS.HSMM	HSMM Skeletal Muscle Myoblasts Cells		19_DNase					DNase
E121	ENCODE 2012	MUS.HSMMT	HSMM cell-derived Skeletal Muscle Myotubes Cells		22_PromP	H3K4me1_Enh		H3K27ac_Enh		DNase
E125	ENCODE 2012	BRN.NHA	NH-A Astrocytes Primary Cells		19_DNase					DNase
E126	ENCODE 2012	SKIN.NHDFAD	NHDF-Ad Adult Dermal Fibroblast Primary Cells		19_DNase					
E128	ENCODE 2012	LNG.NHLF	NHLF Lung Fibroblast Primary Cells							DNase

Supplementary Table S9. Hits from selected eQTL studies from HaploReg.[2]

**rs2486963 (*CHIT1*)**				
**Study ID**	**PMID**	**Tissue**	**Correlated gene**	**p-value**
GTEx2015_v6	25954001	Whole Blood	CHIT1	4.43E-06
Westra2013	24013639	Whole Blood	MYBPH	7.10E-33
**rs2244385 (*CHIT1*)**				
GTEx2015_v6	25954001	Brain Cortex	CHIT1	3.81E-07
GTEx2015_v6	25954001	Whole Blood	CHIT1	2.03E-06
**rs1669691 (1.2kb 5' of *TDRP*)**				
GTEx2015_v6	25954001	Cells transformed fibroblasts	TDRP	1.83E-10
**rs1669707 (1.9kb 5' of *TDRP*)**				
GTEx2015_v6	25954001	Adipose Subcutaneous	TDRP	8.00E-06
GTEx2015_v6	25954001	Cells transformed fibroblasts	TDRP	2.19E-10
**rs6993769 (6.2kb 3' of *ERICH1*)**				
Westra2013	24013639	Whole Blood	ERICH1	1.02E-162
**rs28393818 (5.8kb 3' of *ERICH1*)**				
No eQTLs reported				
**rs7238797 (*DLGAP1*)**				
No eQTLs reported				

Supplementary Table S10. Details of overlapping regulatory altered motifs.[2] The sequence of regulatory motifs is annotated using the International Union of Pure and Applied Chemistry (IUPAC). It has defined a standard representation of DNA bases by single characters that specify either a single base (e.g., G for guanine, A for adenine) or a set of bases (e.g., R for either G or A). UCSC uses these single-character codes to represent multiple observed alleles of single-base polymorphisms.

**rs2486963 (*CHIT1*)**				
**Position Weight Matrix ID**	**Strand**	**Ref**	**Alt**	Ref: GCAAAGCTCACTGTGCTGCCCTCCAGCTG**A**GGTGCCAAGAACCCAGAAGGAAATTCAGC
				Alt: GCAAAGCTCACTGTGCTGCCCTCCAGCTG**G**GGTGCCAAGAACCCAGAAGGAAATTCAGC
AP-4_2	+	14.2	3.6	NVSMGCTGHB
AhR_1	+	-4.3	7.6	SYYSVRRSTHGCGTGASW
CTCF_disc7	+	8.8	11.5	CCRSCWGGGG
HEY1_disc2	-	7.7	11.1	SSNSSSSNSNNSSNNS
LBP-1_2	+	1	11.6	CVGCTKS
Lmo2-complex_1	-	11.1	13.1	VDVCASCTGBVS
Nanog_disc3	-	11.5	12.2	CCWGCWGDGV
Rad21_disc6	-	11.1	11.4	VSYDSSMNSNNSNDSNS
SREBP_known2	-	0.8	12.7	TGGSGTG
**rs2244385 (*CHIT1*)**				
**Position Weight Matrix ID**	**Strand**	**Ref**	**Alt**	Ref: GTCTTTTTTCCCATGAGGGCCTCGGGGCT**C**AAAAGAAGCCACCAAACAGGGCCTGCTGG
				Alt: GTCTTTTTTCCCATGAGGGCCTCGGGGCT**G**AAAAGAAGCCACCAAACAGGGCCTGCTGG
AP-2_disc2	-	13.4	13.2	NNSCCYCAGGSMHND
AP-2_known1	+	12.5	13.1	VGCCBSVGGVVBV
BCL_disc5	-	6.6	10.2	CTGATAAG
**rs1669691 (1.2kb 5' of *TDRP*)**				
**Position Weight Matrix ID**	**Strand**	**Ref**	**Alt**	Ref: GGGCAGCACCCCTCCCACATGCCTCCCTC**C**CCCGGTGACCGCTAAACCTACCGGCCGCA
				Alt: GGGCAGCACCCCTCCCACATGCCTCCCTC**G**CCCGGTGACCGCTAAACCTACCGGCCGCA
AP-2_known2	+	8.3	12.3	NNYNYHGCYYRSSGVB
CAC-binding-protein	-	13	1	SCCWSCCYY

CTCF_disc8	-	11.2	5.8	SCNBYNBSYDSYNS
ERalpha-a_disc4	-	10.2	10.1	SHBSNSNSNSCHNS
EWSR1-FLI1	-	-4.9	-16.8	CCTTCCTTCCTTCCTTCC
MAZ	-	14.5	2.6	CCCTCCCY
Pax-5_known3	-	10.5	-1.5	VSDSYSKCYMYKCWWCVBTGMSYYWSGV
Rad21_disc6	-	12.1	11.3	VSYDSSMNSNNSNDSNS
SP1_known1	-	11.9	6.4	CCCCKYCYMC
STAT_disc7	-	13	13	BHCHBYCYBNCYC
TFII-I	-	14.6	3.9	CCTMMVYCH
WT1	+	11.4	3.9	SVCHCMBSS
Znf143_disc3	-	11.7	11.7	VCYVCVNBBCCCVSVVBSC
**rs1669707 (1.9kb 5' of *TDRP*)**				
**Position Weight Matrix ID**	**Strand**	**Ref**	**Alt**	Ref: ATTTTGAGAAGAACTTTCCCGAGTACTAT**G**AATTTGACTCAGTTCATTCATTAGCTCCT
				Alt: ATTTTGAGAAGAACTTTCCCGAGTACTAT**C**AATTTGACTCAGTTCATTCATTAGCTCCT
AP-1_disc4	-	-4.4	7.6	TYTCWNWWTKAST
Foxa_disc4	-	13.2	1.2	AKGAAYTT
STAT_disc5	-	12.8	0.9	NAKGAAYTT
**rs6993769 (6.2kb 3' of *ERICH1*)**				
**Position Weight Matrix ID**	**Strand**	**Ref**	**Alt**	Ref: CCCAAGGGGCATCCAGGAGGGGCCCCACC**C**GATCCCCACGGACCATGTTTGAGAGAGAG
				Alt: CCCAAGGGGCATCCAGGAGGGGCCCCACC**T**GATCCCCACGGACCATGTTTGAGAGAGAG
CACD_2	-	11.7	7.8	CMMCRCCC
DEC	+	0.3	12.3	VBBCAHSTGMDBN
EBF_disc2	-	10.4	11.3	SHSBNBCCHNRRS
HEN1_1	-	6.6	18.6	HDRGGVMGCAGCTGMKNCCHHH
LXR_1	+	1.5	4.3	TGMMCBSBRGTRACCCYR
MAZR	-	14.6	12.4	KKDCCCCCCCCSH
Mxi1_known1	+	0.3	12.2	BCACVTGNYB

PLAG1	-	8.1	5.3	CCCCCKWKGSCCCC
ZEB1_disc1	-	0.7	12.7	YHYACCTG
**rs28393818 (5.8kb 3' of *ERICH1*)**				
**Position Weight Matrix ID**	**Strand**	**Ref**	**Alt**	Ref: GTGGGGTGGTCAGGCCAGAGCCCCGGAAG**G**GGGTGGAGACACAAGGCCCCGAGTCCAGT<br>Alt: GTGGGGTGGTCAGGCCAGAGCCCCGGAAG**C**GGGTGGAGACACAAGGCCCCGAGTCCAGT
BCL_disc1	+	3.4	13.1	RSCGGAAGYG
BDP1_disc1	+	-3	-2	CMNGGMRGRCTKCCTGGAGGAGG
CACD_2	+	11.5	0.6	GGGYGKKG
CCNT2_disc2	-	12.6	9.1	RGGGBHGGGG
CTCF_disc8	+	12	12.1	SNRSHRSVNRVNGS
ELF1_disc1	-	2.4	12.2	WMSCGGAAGY
ELF1_disc2	+	11	11.8	SSVSGMVSBS
Ets_disc2	-	4	10.6	RSCGGAAGTS
Ets_known3	+	14.1	15.3	VSCGGAAGYGSR
Ets_known5	+	12.5	15.3	SCGGAAGYGVN
Irf_disc4	+	12.1	0.8	RRKRGGCGKRGCYDV
SP1_disc3	+	14.1	2.6	SBNDGRRGGMRGGRS
SP1_known2	+	11.9	8.6	VVDGGGMGGRGBB
Sp4	-	12.8	3	VNNRRGGGGGCGKDDHV
Zfp281	-	11.5	4.4	GRKGGGGGRKGGKVH
Zfp740	-	12.6	3.6	NRNDKKGGGGKGGNNR
**rs7238797 (*DLGAP1*)**				
**Position Weight Matrix ID**	**Strand**	**Ref**	**Alt**	Ref: AAACTAGTGATCTTCAGACAGCATTCCTC**T**GTCCACGGTCCTCACCGCGAGGCAGCCCC<br>Alt: AAACTAGTGATCTTCAGACAGCATTCCTC**C**GTCCACGGTCCTCACCGCGAGGCAGCCCC
BCL_disc3	-	15.7	3.8	TGTCCRTGGT
NRSF_disc2	+	16.1	4.1	TGTCCRTGGT
NRSF_disc4	-	12.5	11.1	SCYSNSCNNSSNSS

NRSF_known1	-	10.9	-0.9	GYRCTGTCCRYGGTGCTGR
NRSF_known3	-	1.3	-10.7	GSYKCTGTCCGYGGTGCTGAA
SPIB	-	11.3	0	TTCCKSW
Sin3Ak-20_disc1	+	10	4.5	TGTCCDYGGTGCTGA
Sin3Ak-20_disc3	-	8.6	-3.4	CTSTCCWTGGT
TEF-1_2	-	14.1	14.6	YRCATTCCWSNB
TFII-I	-	1.3	13	CCTMMVYCH

Supplementary Table S11. Copy Number Variations (CNVs) filtering and analysis details.[7-9]

Analysis Step	Description	Count
**1. Initial CNV Detection**	Total CNVs Identified (Raw PennCNV Output)	28,114
	Total SNPs within all CNVs	427,313
**2. Sample-Level Quality Control**	**Criteria: Sample LRR_SD < 0.25 and NumCNV < 1000**	
	CNVs Remaining Post-Sample QC	19,849
	SNPs Lost during Sample QC	154,546
**3. CNV-Level Quality Filters**	**Criteria: Size (10kb–1Mb) and SNP Count (>10)**	
	Input CNVs for Filtering	19,849
	CNVs removed due to small size (< 10 kb)	9,942
	CNVs removed due to large size (> 1 Mb)	12
	CNVs removed due to low SNP count (≤ 10)	12,461
	Total unique CNVs removed by any filter	13,604
	CNVs Remaining Post-Filters	6,245
**4. Merging Adjacent CNVs**	**Criteria: Gap ≤ 20% of combined length**	
	CNVs Pre-Merge	6,245
	Number of CNVs reduced by merging	470
	Final High-Confidence CNV Set	5,775

Supplementary Table S12. Cohort characteristics and Copy Number Variations (CNVs) descriptive statistics in *chlamydia trachomatis* (*Ct*) reinfection cases and controls.

Category	Metric (units)	Cases	Controls
**Cohort overview**	Samples (n)	60	225
	Total CNVs (events)	1725	4050
	CNVs per person, mean	28.75	18
	CNVs per person, median	8	8
	Total CNV length, sum of event lengths (bp)	119885134	234715718
	CNV length per event, median (bp)	34067	31902
**CNV type distribution**	Deletions, n (%)	691(40.1%)	2669(65.9%)
	Duplications, n (%)	1034(59.9%)	1381(34.1%)
**Event size (by CNV type)**	Deletions, mean length (bp)	52112	50913
	Deletions, median (P25, P75) length (bp)	30466(19446,57827)	30478(19651,52080)
	Duplications, mean length (bp)	81118	71563
	Duplications, median (P25, P75) length (bp)	36924(21597,70654)	35817(20536,74893)
**Call confidence (by CNV type)**	Deletions, mean (median, SD) confidence	52.45(31.29,81.76)	54.96(31.12,91.37)
	Duplications, mean (median, SD) confidence	46.68(28.62,56.82)	65.25(35.8,90.3)
**Event size bins (counts, %)**	Deletions, 10–100 kb, n (%)	615(89%)	2385(89.4%)
	Deletions, 100 kb–1 Mb, n (%)	76(11%)	283(10.6%)
	Deletions, >1 Mb, n (%)	0(0%)	1(0%)
	Duplications, 10–100 kb, n (%)	870(84.1%)	1136(82.3%)
	Duplications, 100 kb–1 Mb, n (%)	157(15.2%)	242(17.5%)
	Duplications, >1 Mb, n (%)	7(0.7%)	3(0.2%)

All results are from gene-body CNV association analyses using hg38/GRCh38 coordinates and UCSC knownGene annotations.
Gene-body CNVs were defined as CNVs overlapping any part of the annotated gene transcript span, including exons and introns.
Section A lists genes with at least one exonic CNV overlap, whereas Section B lists additional genes with intronic-only CNV overlap and no annotated exon overlap in this dataset.
Deletions and duplications were analyzed separately.
Case and control carriers refer to individuals with ≥1 CNV overlapping the indicated gene-body annotation.

Supplementary Table S13. Gene-based Copy Number Variations (CNVs) Association Results (gene-body analysis; exonic hits listed first).

Section A. Exonic CNV hits (subset of gene-body analysis)				
**Section A1. Exonic deletions (DEL)** (Total cases = 55 ; Total controls = 198)				
**Gene**	**Case Carriers**	**Control Carriers**	**OR [95% CI]**	**P-value**
ATAD3A	3 (5.45%)	1 (0.51%)	8.7778 [1.4086,91.9923]	0.0203
CARD14	3 (5.45%)	1 (0.51%)	8.7778 [1.4086,91.9923]	0.0203
TMEM240	3 (5.45%)	1 (0.51%)	8.7778 [1.4086,91.9923]	0.0203
**Section A2. Exonic duplications (DUP)** *(Total cases = 54 ; Total controls = 203)*				
**Gene**	**Case Carriers**	**Control Carriers**	**OR [95% CI]**	**P-value**
AC007292.3	3 (5.56%)	1 (0.49%)	9.1748 [1.4720,96.1684]	0.0180
AC007292.4	3 (5.56%)	1 (0.49%)	9.1748 [1.4720,96.1684]	0.0180
ANO9	3 (5.56%)	1 (0.49%)	9.1748 [1.4720,96.1684]	0.0180
BAIAP2-AS1	3 (5.56%)	1 (0.49%)	9.1748 [1.4720,96.1684]	0.0180
CTD-2561B21.10	3 (5.56%)	1 (0.49%)	9.1748 [1.4720,96.1684]	0.0180
CTD-2561B21.11	3 (5.56%)	1 (0.49%)	9.1748 [1.4720,96.1684]	0.0180
GPSM1	3 (5.56%)	1 (0.49%)	9.1748 [1.4720,96.1684]	0.0180
LA16c-359F1.1	3 (5.56%)	1 (0.49%)	9.1748 [1.4720,96.1684]	0.0180
RAB11FIP3	3 (5.56%)	1 (0.49%)	9.1748 [1.4720,96.1684]	0.0180
RP11-386I8.4	3 (5.56%)	1 (0.49%)	9.1748 [1.4720,96.1684]	0.0180
RP11-386I8.5	3 (5.56%)	1 (0.49%)	9.1748 [1.4720,96.1684]	0.0180
SIGIRR	3 (5.56%)	1 (0.49%)	9.1748 [1.4720,96.1684]	0.0180
TPPP	4 (7.41%)	3 (1.48%)	5.1047 [1.2053,23.4798]	0.0278
ARHGAP39	3 (5.56%)	2 (0.99%)	5.4777 [1.0384,33.6327]	0.0453
LA16c-381G6.1	3 (5.56%)	2 (0.99%)	5.4777 [1.0384,33.6327]	0.0453
PIEZO1	3 (5.56%)	2 (0.99%)	5.4777 [1.0384,33.6327]	0.0453
PKP3	3 (5.56%)	2 (0.99%)	5.4777 [1.0384,33.6327]	0.0453
PLA2G4F	3 (5.56%)	2 (0.99%)	5.4777 [1.0384,33.6327]	0.0453
RP11-1391J7.1	3 (5.56%)	2 (0.99%)	5.4777 [1.0384,33.6327]	0.0453
RP13-122B23.8	3 (5.56%)	2 (0.99%)	5.4777 [1.0384,33.6327]	0.0453

SHC2	3 (5.56%)	2 (0.99%)	5.4777 [1.0384,33.6327]	0.0453
VPS39	3 (5.56%)	2 (0.99%)	5.4777 [1.0384,33.6327]	0.0453
ZNF140	3 (5.56%)	2 (0.99%)	5.4777 [1.0384,33.6327]	0.0453
ZNF709	3 (5.56%)	2 (0.99%)	5.4777 [1.0384,33.6327]	0.0453
**Section B. Additional gene-body CNV hits (intronic only; no exonic overlap)**				
**Section B1. Gene-body deletions (intronic only)** *(Total cases = 57 ; Total controls = 216)*				
**Gene**	**Case Carriers**	**Control Carriers**	**OR [95% CI]**	**P-value**
FAM166A	3 (5.26%)	2 (0.93%)	5.5101 [1.0461,33.7882]	0.0444
SSU72	3 (5.26%)	2 (0.93%)	5.5101 [1.0461,33.7882]	0.0444
**Section B2. Gene-body duplications (intronic only)** *(Total cases = 55 ; Total controls = 216)*				
**Gene**	**Case Carriers**	**Control Carriers**	**OR [95% CI]**	**P-value**
SNORA1	7 (12.73%)	9 (4.17%)	3.3776 [1.1950,9.2495]	0.0228
SNORA63	9 (16.36%)	14 (6.48%)	2.8532 [1.1518,6.7997]	0.0245
CTD-2192J16.20	4 (7.27%)	4 (1.85%)	4.1262 [1.0330,16.4951]	0.0452
CTD-2192J16.21	4 (7.27%)	4 (1.85%)	4.1262 [1.0330,16.4951]	0.0452
ZNF564	4 (7.27%)	4 (1.85%)	4.1262 [1.0330,16.4951]	0.0452
LINC00864	10 (18.18%)	19 (8.80%)	2.3373 [1.0022, 5.2097]	0.0494
U1	10 (18.18%)	19 (8.80%)	2.3373 [1.0022, 5.2097]	0.0494

All results are from gene-body analysis (exons + introns).
Rows in Section A represent genes with at least one exonic CNV.
Rows in Section B represent additional genes for which CNVs overlap intronic regions only (no exonic overlap in this dataset).
Case and control carriers refer to individuals with ≥1 CNV overlapping the gene body.

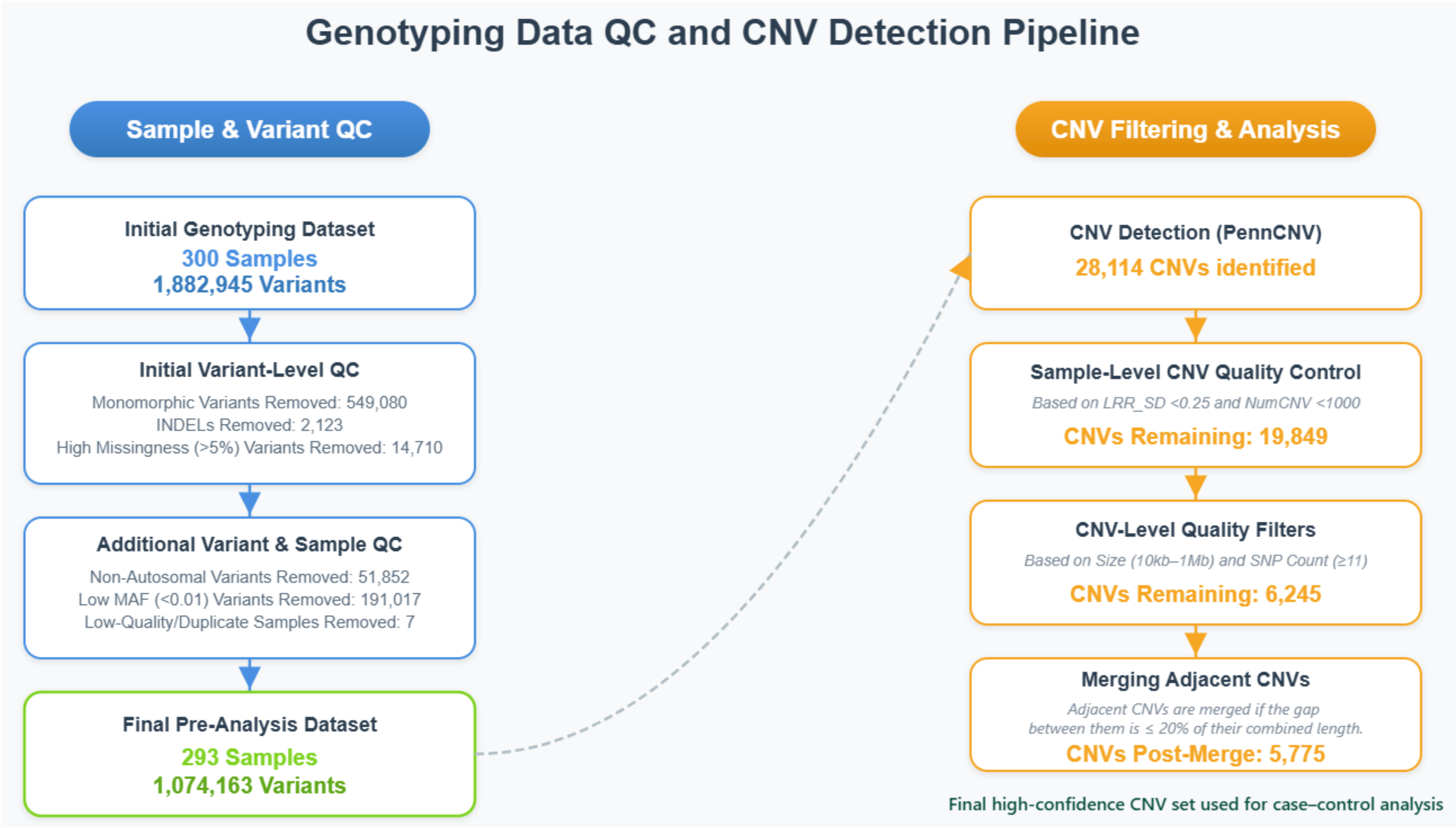

Supplementary Figure S1: Genotyping data quality control and copy number variation determination analysis pipeline.

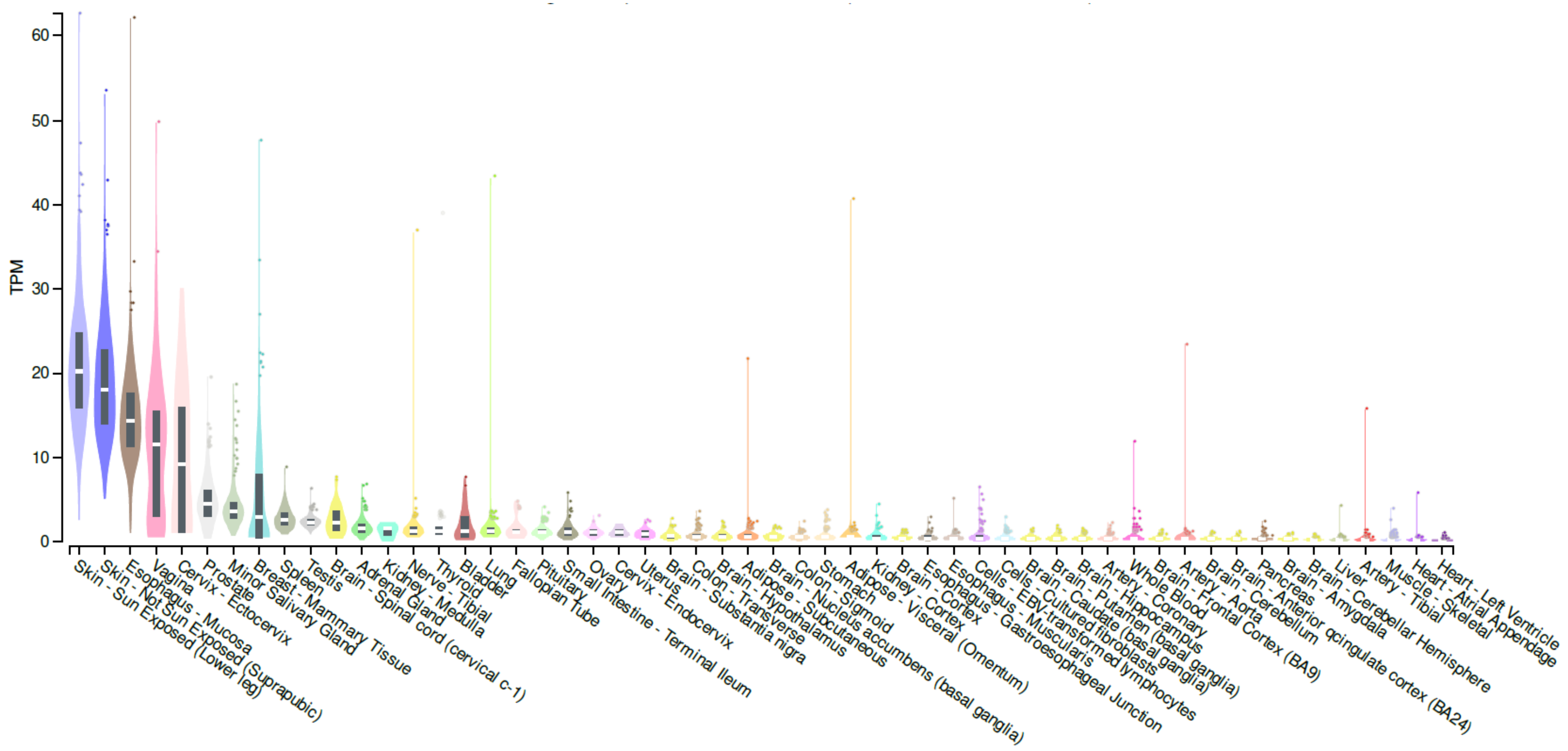

Supplementary Figure S2: Caspase recruitment domain family member 14 (*CARD14*) gene expression in 54 tissues, using GTEx Analysis Release V10 (dbGaP Accession phs000424.v10.p2).[10]

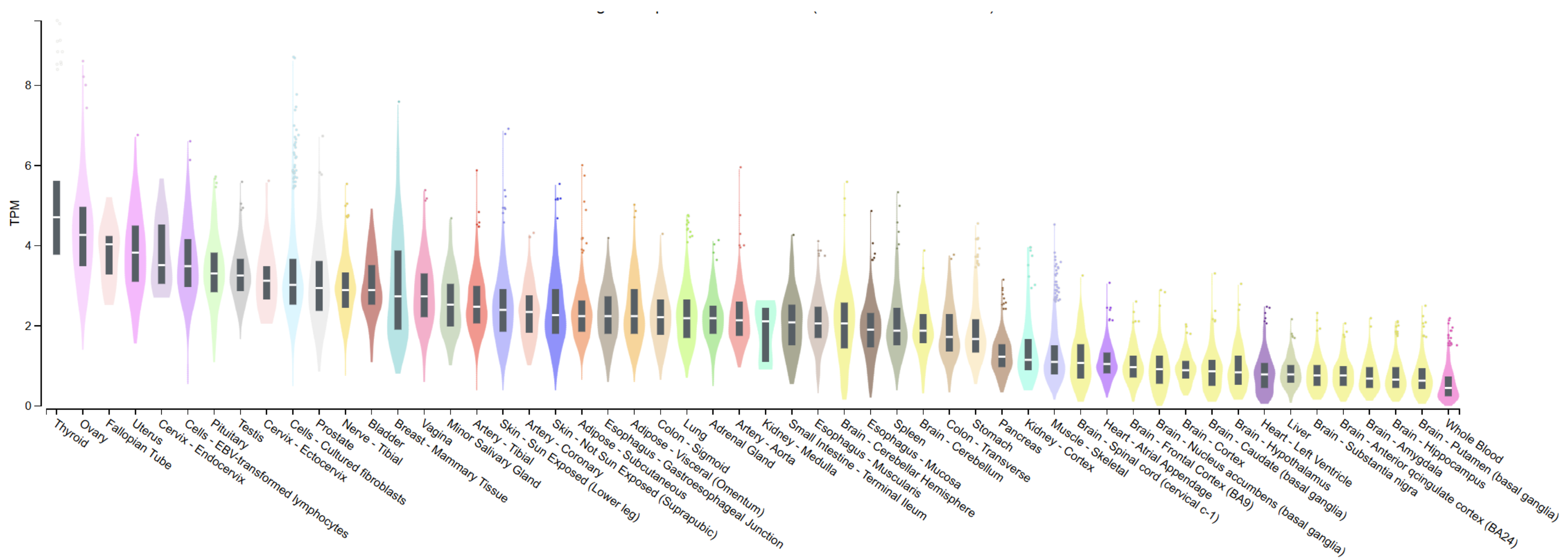

Supplementary Figure S3: Zinc Finger Protein 140 (*ZNF140*) gene expression in 54 tissues, using GTEx Analysis Release V10 (dbGaP Accession phs000424.v10.p2).[10]

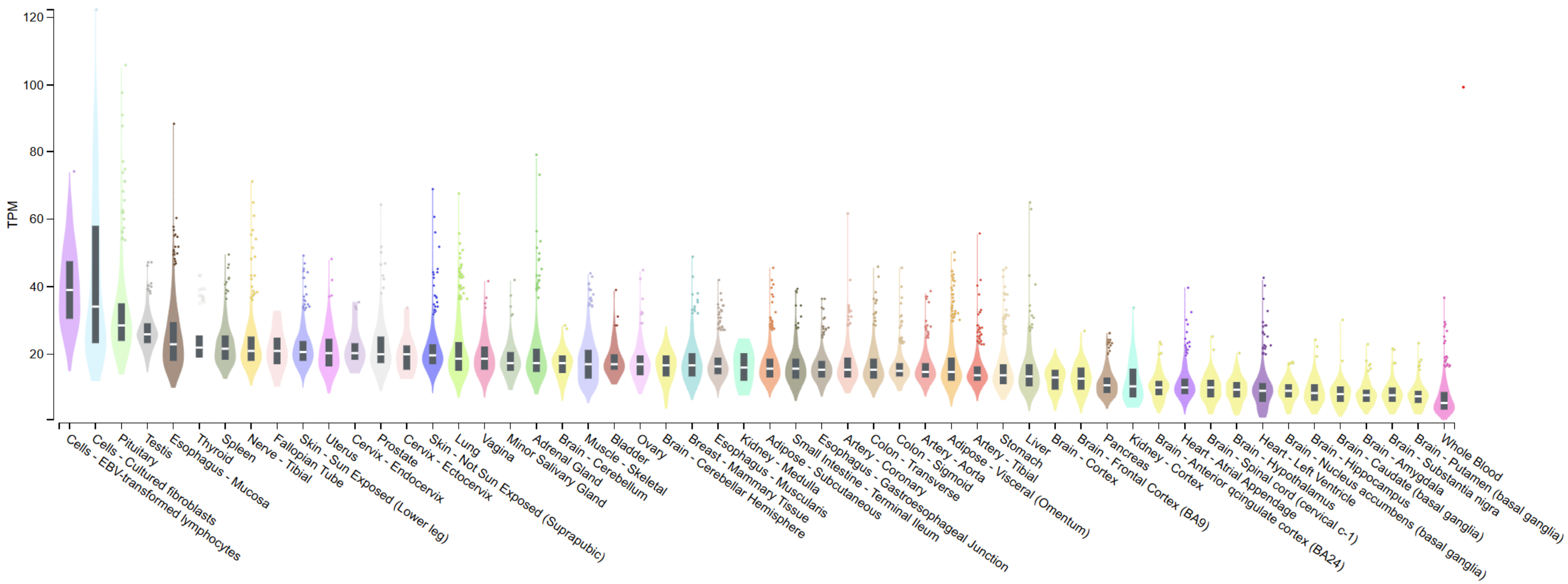

Figure S4: ATPase family AAA domain-containing protein 3A (*ATAD3A*) gene expression in 54 tissues, using GTEx Analysis Release V10 (dbGaP Accession phs000424.v10.p2).[10]

1. Tiwari, H.K., et al., *Genome-wide association study of chlamydia reinfection in African American women.* Front Immunol, 2025. **16**: p. 1594317.
2. Ward, L.D. and M. Kellis, *HaploReg v4: systematic mining of putative causal variants, cell types, regulators and target genes for human complex traits and disease.* Nucleic Acids Res, 2016. **44**(D1): p. D877–81.
3. Boyle, A.P., et al., *Annotation of functional variation in personal genomes using RegulomeDB.* Genome Res, 2012. **22**(9): p. 1790–7.
4. Schaub, M.A., et al., *Linking disease associations with regulatory information in the human genome.* Genome Res, 2012. **22**(9): p. 1748–59.
5. Breeze, C.E., et al., *FORGEdb: a tool for identifying candidate functional variants and uncovering target genes and mechanisms for complex diseases.* Genome Biol, 2024. **25**(1): p. 3.
6. Guo, L. and J. Wang, *rSNPBase 3.0: an updated database of SNP-related regulatory elements, element-gene pairs and SNP-based gene regulatory networks.* Nucleic Acids Res, 2018. **46**(D1): p. D1111–d1116.

7. Wang, K., et al., *PennCNV: an integrated hidden Markov model designed for high-resolution copy number variation detection in whole-genome SNP genotyping data*. Genome Res, 2007. **17**(11): p. 1665–74.
8. Diskin, S.J., et al., *Adjustment of genomic waves in signal intensities from whole-genome SNP genotyping platforms*. Nucleic Acids Res, 2008. **36**(19): p. e126.
9. Wang, K., et al., *Modeling genetic inheritance of copy number variations*. Nucleic Acids Res, 2008. **36**(21): p. e138.
10. *Human genomics. The Genotype-Tissue Expression (GTEx) pilot analysis: multitissue gene regulation in humans*. Science, 2015. **348**(6235): p. 648–60.
